# Chemical and Genetic Validation of an Essential Calcium Entry Channel of Trypanosoma brucei as a Therapeutic Target

**DOI:** 10.1021/acsinfecdis.5c00329

**Published:** 2025-06-03

**Authors:** Guozhong Huang, Harmanpreet Singh, Priti Singh, Rohit Kumar Varshnaya, Donald Hamelberg, Binghe Wang, Roberto Docampo

**Affiliations:** † Center for Tropical and Emerging Global Diseases and Department of Cellular Biology, 1355 University of Georgia, Athens, Georgia 30602, United States; ‡ Department of Chemistry, 1373 Georgia State University, Atlanta, Georgia 30303, United States

**Keywords:** calcium channel, docking analysis, target validation, *Trypanosoma brucei*, yeast complementation

## Abstract

The Trypanosoma brucei group of parasites causes Nagana in cattle and human African trypanosomiasis, or sleeping sickness, in humans. Current drugs against these parasites have severe toxicity, vaccines are not available, and development of drug resistance makes finding new chemotherapeutic targets imperative. Ion channels, which are involved in several biological processes, are targets of many therapeutically useful agents, and they remain significantly underexplored as therapeutic targets in parasites. Here, we report the presence of a voltage gated Ca^2+^ channel (VGCC, TbCa_v_), which is localized in the flagellar plasma membrane (PM) of T. brucei and is essential for proliferation of both bloodstream (BSF) and procyclic forms (PCF) of the parasite. TbCa_V_ is a single subunit channel capable of transporting Ca^2+^ when expressed in mutant yeast lacking PM Ca^2+^ channels or in HEK293T cells. Through the virtual screening of a commercial chemical library using dynamic ensembles of various conformations of TbCa_v_ and associated docking analyses, several inhibitors of TbCa_v_ were discovered. As pharmacological validation of the essential roles of TbCa_v_, these compounds were shown to inhibit T. brucei growth with the most potent agent, *N*-(7-nitro-2,1,3-benzoxadiazol-4-yl) acetamide (NBD-A), exhibiting an EC_50_ of 25 ± 3 nM and no cytotoxicity in Vero cells possessing related channels. Thus, such studies constitute pharmacological validation of TbCa_v_ as a viable therapeutic target of T. brucei.

## Graphical abstract

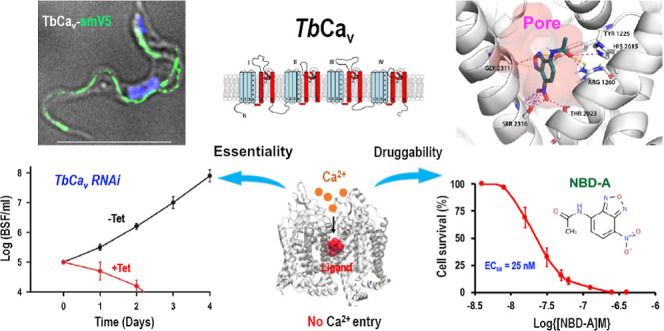

The Trypanosoma brucei group of parasites is the causative agent of sleeping sickness, human African trypanosomiasis (HAT), and Nagana in cattle. According to the World Health Organization, this neglected disease occurs in 36 sub-Saharan African countries where millions are at risk of infection, has had an incidence of thousands of reported cases per year and many reported and unreported deaths. Chemotherapy remains unsatisfactory especially for advanced cases.^1^ A recently approved drug (fexinidazole) can be used orally except for patients with advanced stage disease and children under 6 years old or weighing less than 20 kg.^2^ It has also been reported that oxaboroles (acoziborole) are orally effective against HAT.^3^ However, the frequent emergence of trypanocide resistance in humans^4^ and animals^5^ has undermined the use of most compounds. African animal trypanosomiasis is also an important economic problem, preventing people from raising cattle and earning a living in vast regions of sub-Saharan Africa. In addition, T. brucei shares the presence of Ca_V_ channels with other trypanosomatids like Trypanosoma cruzi, the agent of Chagas disease, and Leishmania spp., agents of leishmaniases. Drugs with different modes of action are needed. Chemical validation of Ca_V_ channels as a therapeutic target could lay a strong foundation for developing novel treatments of this related trypanosomiasis.

Ca^2+^ is a universal signaling ion.^6^ Ca^2+^ through plasma membrane channels or Ca^2+^ release from intracellular stores leads to the stimulation of a variety of physiological responses. No genes in the T. brucei genome encode homologues to various types of plasma membrane Ca^2+^ channels such as store-operated channel (Orai) and the ER Ca^2+^ sensor protein (STIM), ligand-operated channels, and second messenger-operated channels.^7^ However, a putative voltage gated Ca^2+^ channel (Ca_V_) was identified in 2011 ^7^ that has flagellar plasma membrane localization.^8^ In this regard, we have shown that T. brucei BSF are depolarized in high K^+^ medium^9^ and this could activate voltage-gated channels causing changes in cytosolic Ca^2+^.^10^

Mammalian Ca_V_ channels from skeletal muscle are complexes of α1, α2, β, γ, and δ subunits.^11^ In contrast, known Ca_V_ channels of unicellular eukaryotes that have been studied so far,^12,13^ and that do not belong to the Opisthokonta supergroup of eukaryotes, which includes animals and fungi, appear to have only the α1 subunit. In this regard, searches with the human Ca_V_ channel accessory subunits β, α–2/δ, and γ sequences in GenBank failed to find protein homologues in trypanosomes. TbCa_V_ differs from the mammalian Ca_V_ channels in the sequence of several determinants of activity regulation.^13^ The four amino acid residues of the selectivity filter of metazoan (EEEE) are substituted by the amino acids QESE in TbCa_V_.^13^ The α–interaction domain (AID) responsible for interaction of Ca_V_β is absent in the T. brucei channel, in agreement with the absence of β subunit.^13^ However, TbCa_V_ shows conservation of the pre-IQ and IQ motif that are important for calmodulin binding.^13^ Taken together, it would be possible to target Ca^2+^ entry in T. brucei through the direct inhibition of the TbCa_V_ channel. It is interesting to note that an ortholog of this channel present in Trypanosoma equiperdum has 99.85% identity to TbCa_V_ ^14^ and is, like the T. cruzi ortholog,^15,16^ activated by sphingosine.

In this work, we describe that knockdown of *TbCa_V_* by RNAi is lethal for both procyclic (PCF) and bloodstream (BSF) forms of T. brucei. In situ epitope tagged TbCa_V_ localizes to the flagellum plasma membrane and its role in Ca^2+^ entry is supported by (1) functional expression of *TbCa*_*V*_ in yeast deficient in plasma membrane Ca^2+^ channels and (2) the increase in Ca^2+^ uptake detected when *TbCa_V_* was expressed in HEK293T cells. Using an innovative approach of examining conformational ensembles to do docking analysis and thus virtual screening, we have screened a commercial library, leading to the identification of several TbCa_V_ inhibitors with the most potent, having EC_50_ of 25 ± 3 nM, capable of killing the parasite without cytotoxicity to mammalian cells possessing VGCCs. Taking into account the considerable functional and evolutionary differences with mammalian Ca_V_ channels, these findings suggest that TbCa_V_ is a viable target for therapeutic intervention against trypanosomatids.

## Results

### Localization of TbCa_V_ in the Flagellar Plasma Membrane

Prole and Taylor initially reported the presence of L-type Ca_V_ channel homologues in the genomes of several trypanosomatids.^7^ The *TbCa*_*V*_ gene (Tb427.10.2880), also named *FS179* ^8^ or *TbCaCh*,^17^ encodes a predicted protein of 2693 aa with a molecular weight of approximately 301 kDa. The orthologues identified in Leishmania major (LmjF.34.0480) and T. cruzi (TcYC6_0112380) shared 44% and 54% amino acid identity, respectively, to TbCa_V_, which shares only 19%–26% identity with human and rabbit L-type Ca_V_1.1 channels (Q13698 and P07293, respectively) (Figure S1). TbCa_V_ contains 22 transmembrane domains, predicted with Protter^18^ and TMHMM^19^ servers, and shows a four-domain structure comparable to the human L-type Ca_V_ channels^7^ (Figure 1A). Previous work on the flagellum proteome of T. brucei found evidence of the presence of TbCa_V_ in the plasma membrane of these organelles,^8^ and detected weak fluorescence of the flagellum of T. brucei BSF expressing the HA-tagged Ca_V_ channel. The fluorescence signal did not reach the tip of the flagellum^8^ and this was verified in PCF cells (Figure S2).

**Figure 1:**
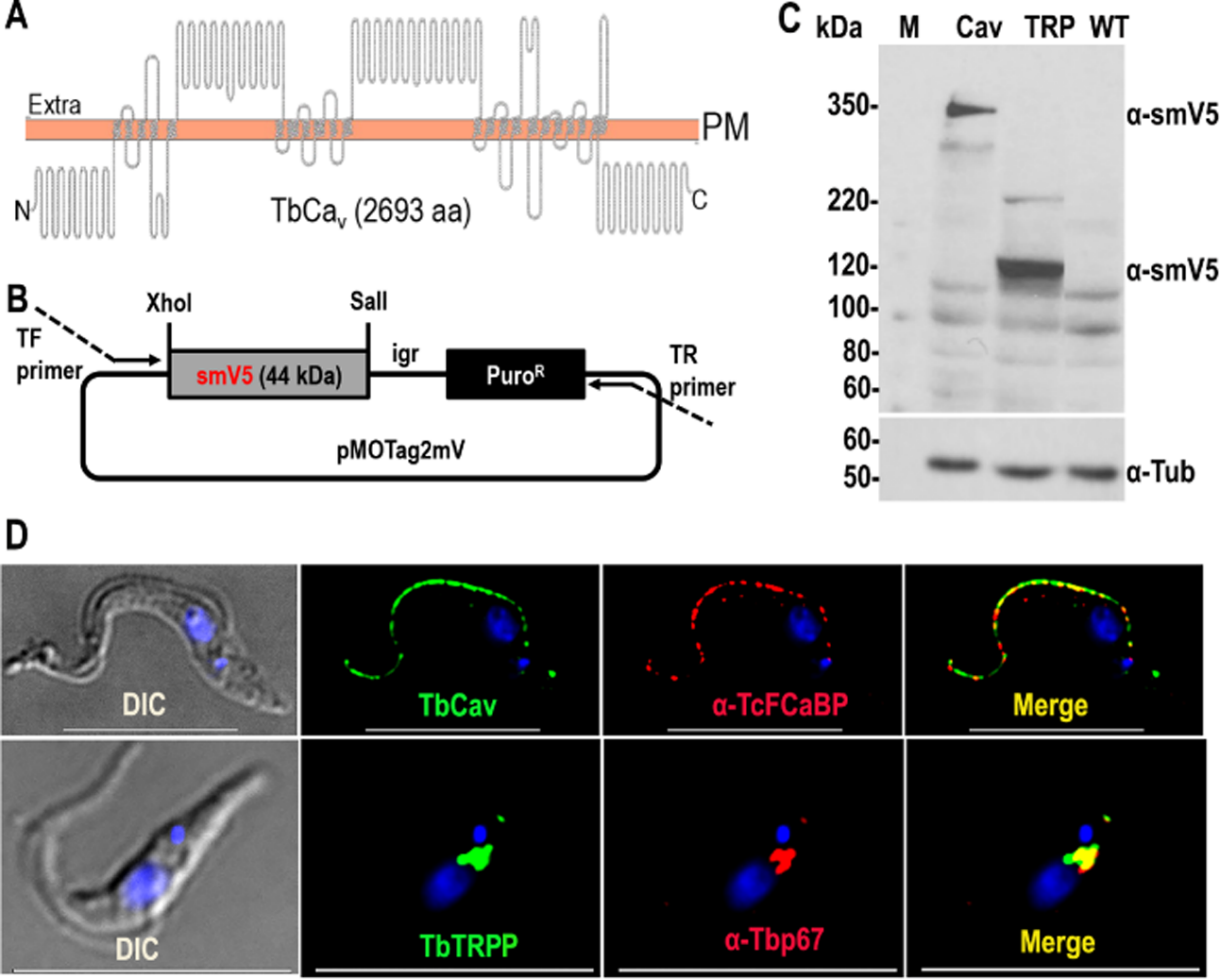
PCR-mediated C-terminal in situ smFP-tagging. (A) Putative topology of TbCa_V_ predicted with Protter. PM, plasma membrane. (B) Map of modified pMOTag2 mV vector. (C) Western blot analysis with anti-V5 Ab shows predicted bands of TbCa_V_-smV5 at 345 kDa and TbTRPP-smV5 at 125 kDa, respectively. Tubulin (α-Tub) was used as loading control. (D) Immunofluorescence analysis (IFA) showing colocalization of TbCa_V_-smV5 with TcFCaBP to the flagellar plasma membrane and TbTRPP-smV5 with Tbp67 to the lysosome of T. brucei PCF, respectively. Scale bars = 10 μm.

To better identify this flagellar localization, we modified the pMOTag vectors^20^ for in situ epitope tagging and tagged the C-terminus of TbCa_V_ with a high-performance tag (spaghetti-monster fluorescence protein [smFP]) with multiple V5 epitope tags^21^ (Figure 1B). We described that this probe enhances the detection of weakly expressed membrane proteins in trypanosomes.^22^ Endogenous tagging of TbCa_V_ with smV5 was confirmed by Western blot analysis using commercial antibodies against V5. Figure 1C shows that a clear band of ∼345 kDa (predicted size) was detected from the TbCa_V_-smV5 cell lysate of T. brucei PCF, compared with the presence of a clear band of ∼125 kDa (predicted size) in cell lysates of cells expressing smV5-tagged TbTRPP (Tb427.08.850, a transient receptor protein channel of T. brucei) and the absence of the bands from the wild type (WT) control. Figure 1D is the IFA of a TbCa_V_-smV5-labeled procyclic form (PCF) cells showing strong labeling that colocalizes with a flagellar plasma membrane marker (antibody TcFCaBP against a T. cruzi flagellar Ca^2+^ binding protein),^23^ compared with the colocalization of TbTRPP-smV5 with the lysosomal marker Tbp67 to the lysosome of T. brucei PCF (Figure 1D).

Figure 2A,B shows that in situ tagged smV5-TbCa_V_ also localizes to the flagellar plasma membrane of T. brucei bloodstream form (BSF) cells. Figure 2C shows the control Western blot analysis of the expression of the protein in two BSF clones.

**Figure 2:**
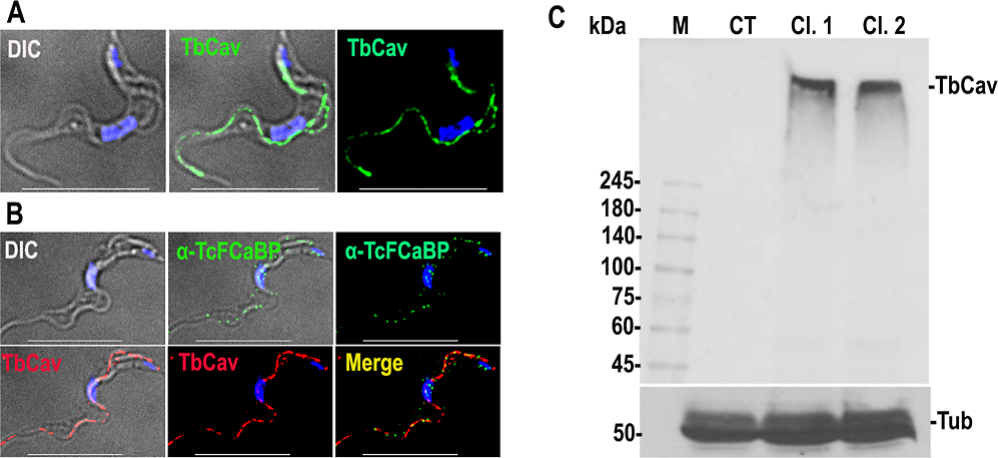
Flagellar localization of TbCa_V_ in BSF cells. (A) IFA showing the flagellar localization of TbCa_V_-smV5. (B) Colocalization with TcFCaBP to the flagellar PM. (C) Western blot analysis with anti-V5 Ab shows predicted bands at 345 kDa in two clones. Tubulin (Tub) was used as loading control. Scale bars = 10 μm.

### Complementation of Yeast Mutants Deficient in Ca^2+^ Influx Channels

To investigate whether *TbCa*_*V*_ encodes a functional Ca^2+^ influx channel, we expressed it in mutant Saccharomyces cerevisiae ELY151 (*cch1::TRP1 mid1::LEU2*) (a gift from Kyle Cunningham^24^). This mutant has deletions of the genes encoding the high-affinity Ca^2+^ influx system (HACS), which consists of two known subunits, Cch1 and Mid1, that are homologous and analogous to the catalytic α-subunits and regulatory α2δ-subunits of mammalian voltage gated calcium channels, respectively.

Figure 3A shows the plasma membrane localization of TbCa_V_ in yeast and Figure 3B shows the Western blot analysis of the protein expression in yeast. This yeast mutant is deficient in mediating the Ca^2+^ entry response to yeast mating pheromone α-factor and does not grow in a halo around a filter paper disc soaked in mating pheromone, compared with wild-type yeast or the *cch1/mid1* mutant expressing *TbCa_V_* (Figure 3C). WT and empty vector controls were used as in.^25,26^ This suggests that TbCa_V_ facilitates the transport of Ca^2+^ in this heterologous system. These results provide functional evidence that TbCa_V_ is a Ca^2+^ entry channel. The ability of TbCa_V_ to complement *cch1/mid1* mutant yeasts also provides evidence that this channel alone, without accessory subunits, can function as a Ca^2+^ channel. In this regard, expression in COS1 cells of the human Ca_V_1.2 channel requires the coexpression of the α1 subunit together with the α2δ and β subunits to show functional expression and plasma membrane localization.^27^

**Figure 3:**
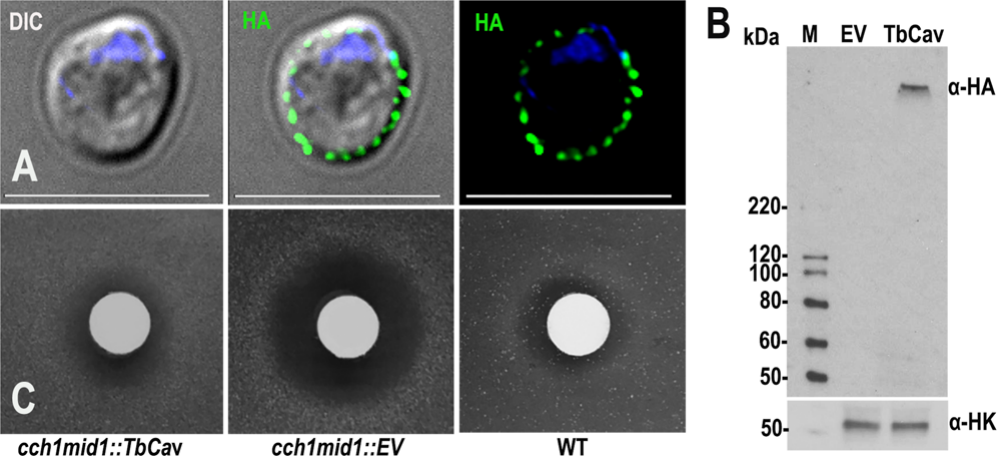
Complementation of yeast mutants deficient in Ca^2+^ channels. (A) IFA showing localization of TbCa_V_-HA in the PM of yeasts. DIC, differential intensity contrast. Scale bars = 10 μm. (B) Western blot analysis showing expression of TbCa_V_-HA (α-HA) in yeast. Anti-HK (α-HK) was used as loading control, EV, empty vector, M, MW markers. (C) TbCa_V_-HA complements growth of the Ca^2+^-uptake deficient yeast *cch1/mid1* mutants. Filter discs containing 10 μg of the mating pheromone α factor inhibit growth in the mutant but not the WT or TbCa_V_-HA-complemented strain. Photographs were taken after 48 h.

### *TbCa_V_* Expression in HEK293T Cells

To further demonstrate the function of TbCa_V_ as a Ca^2+^ entry channel, we cloned *TbCa_V_* into the pcDNA3-1 plasmid and used it to transfect human embryonic kidney cells transformed by the SV40 large T-antigen (HEK293T). HEK293T cells do not express endogenous Ca_V_ channels, but Ca_V_ channels can be expressed exogenously at high levels in these cells.^28^

Figure 4A,B shows that pcDNA-*TbCa_V_-HA* transfected HEK293T cells have increased Ca^2+^ entry (red) further supporting the role of TbCa_V_ as a Ca^2+^ entry channel. Figure 4C is a control Western blot.

**Figure 4:**
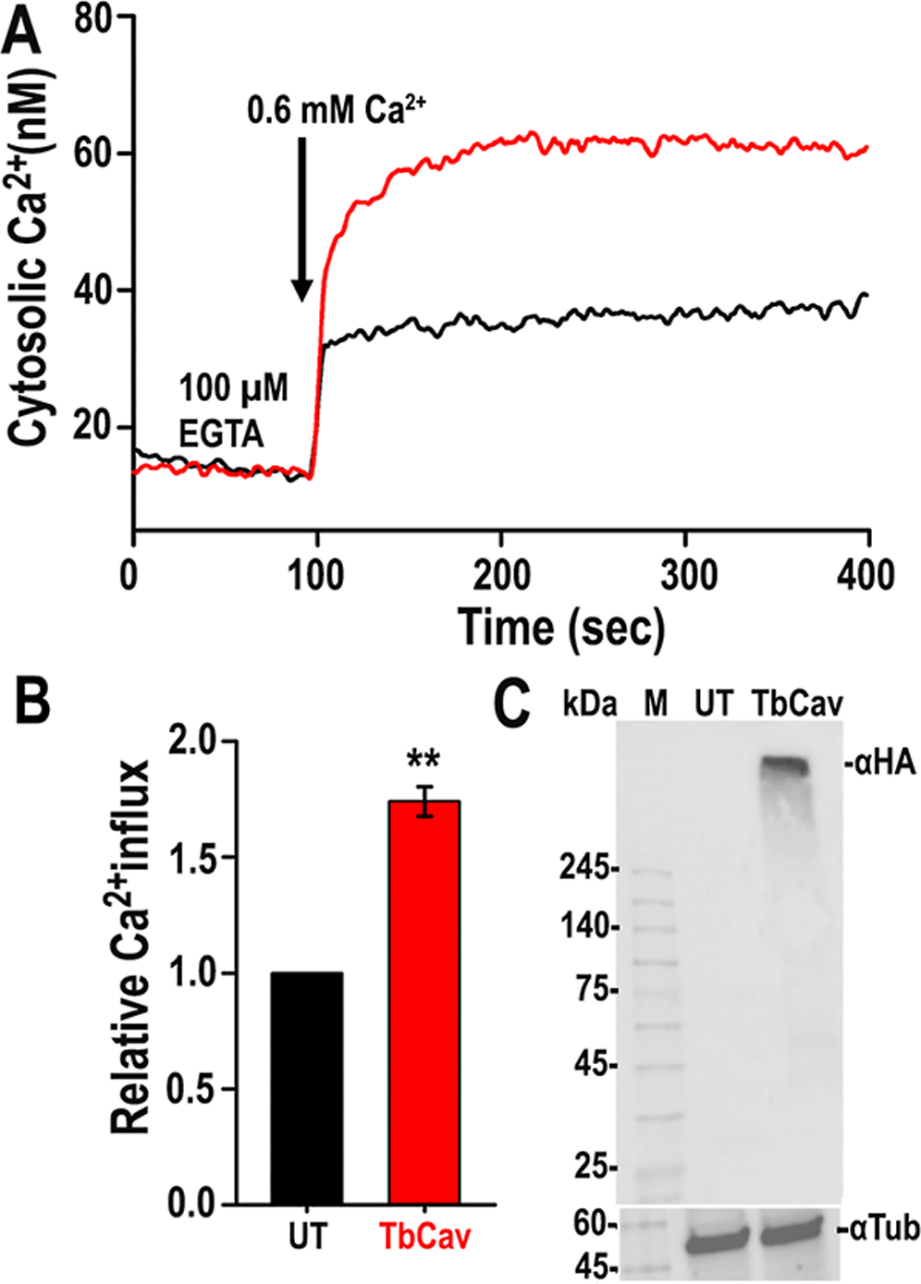
TbCa_v_ promotes Ca^2+^ influx in HEK293T cells. (A,B) Addition of 0.6 mM Ca^2+^ increased the [Ca^2+^]_c_ of Fura 2/AM loaded HEK293T cells by 74% when *TbCa_v_* was transiently transfected with pcDNA3.1-*TbCa_v_-HA* (1.2 μg DNA/well) using GeneJuice (Novagen). 100 μM EGTA was added to remove contaminating Ca^2+^. (B) Relative Ca^2+^ influx. UT, untransfected; TbCa_v_, transfected. (C) Western blot analysis showing expression of TbCa_v_-HA in HEK293T, antitubulin as loading control; M, MW markers. **P < 0.01, *n* = 3. Student’s *t*-test.

### Essentiality of TbCa_V_ for Ca^2+^ Influx and Survival

Knockdown of *TbCa_V_* by the induction of double-stranded RNA resulted in growth defects in both BSF (Figure 5A) and PCF (Figure 5B) cells. The effects were more pronounced with BSF cells with no viable cells after 2 days, as reported before,^8^ while PCF cells survived until day 6. Western blot analyses revealed a correlative decrease in *TbCa_V_* expression in both BSF (Figure 5C) and PCF cells (Figure 5D), respectively. Knockdown (KD) of *TbCa_V_* in BSF cells by RNAi was previously shown to result in flagellar detachment^8^ and this was confirmed in both BSF (Figure 5E–G) and PCF cells (Figure S3).

**Figure 5:**
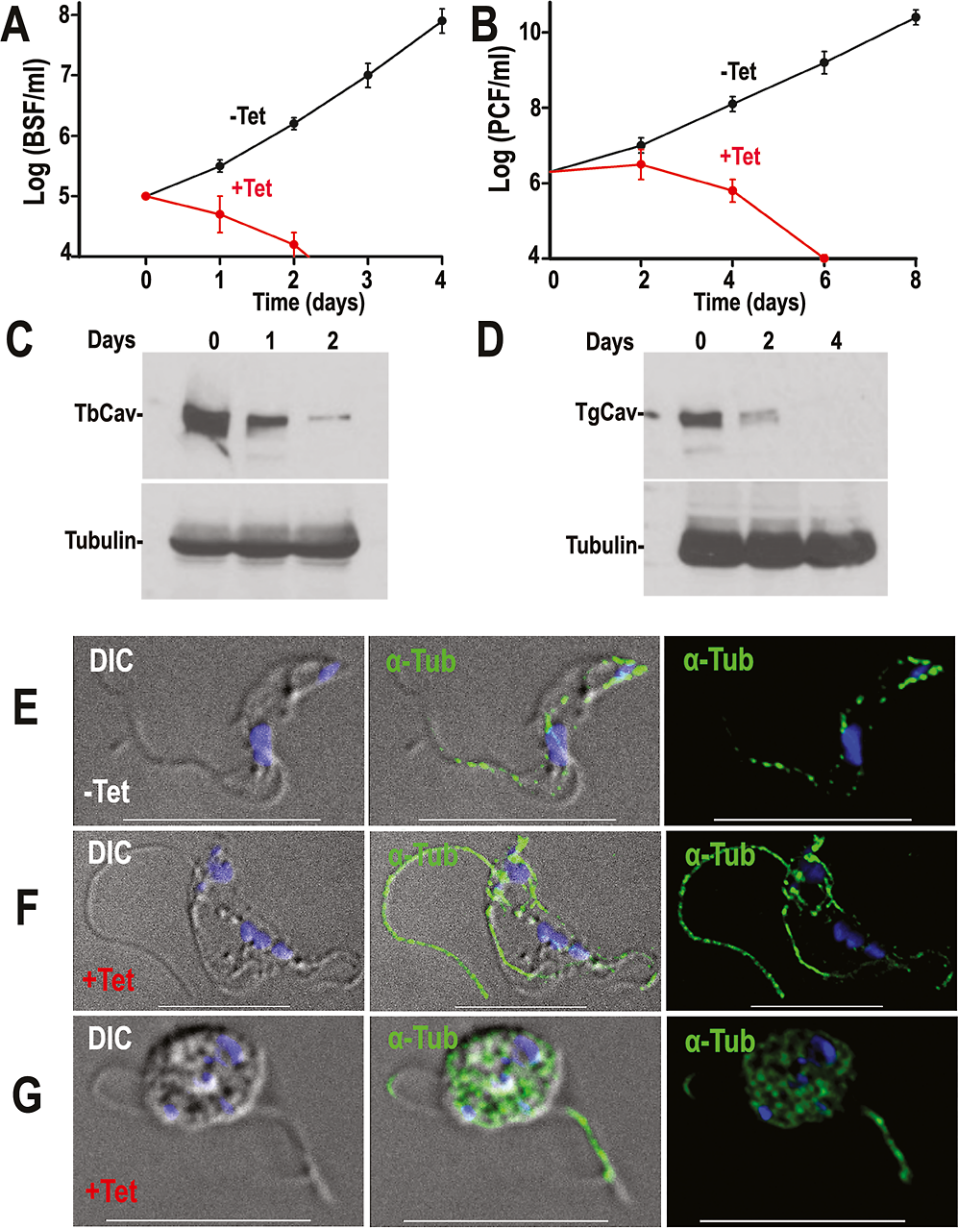
Downregulation of *TbCa_v_* by RNAi affects growth in BSF and PCF cells. (A,B) Growth of BSF (A) and PCF (B) cells in the absence (black line, −Tet) or presence (red line, +Tet) of 1 μg/mL tetracycline for the indicated number of days. (C,D) Western blot analyses of *TbCa_V_* RNAi of BSF (C) and PCF (D) cells grown in the absence (0) or presence (1–2; 2–4) of tetracycline. Total lysates (30 μg) were probed with Ab against tagged TbCa_V_. Tubulin was used as loading control. (E-G) IFA of T. brucei BSF *TbCav*-RNAi cells grown in the absence (Tet−) or presence (Tet+) of 1 μg/mL tetracycline for 20 h. Cells are stained with α-tubulin and DAPI. RNAi cells are multinucleated, multiflagellated and have flagellar detachment from the cell body. Left images are differential intensity contrast. Scale bars = 10 μm.

We then investigated whether the RNAi of *TbCa_V_* affected Ca^2+^ entry. We used the RNAi BSF cell mutants, which were grown either in the absence of tetracycline (control) or with tetracycline for 2 days, when *TbCa_V_* expression was greatly reduced (Figure 5C). After washing, BSF cell lines were loaded with Fura-2AM to study cytosolic Ca^2+^ changes (Figure 6A).

**Figure 6:**
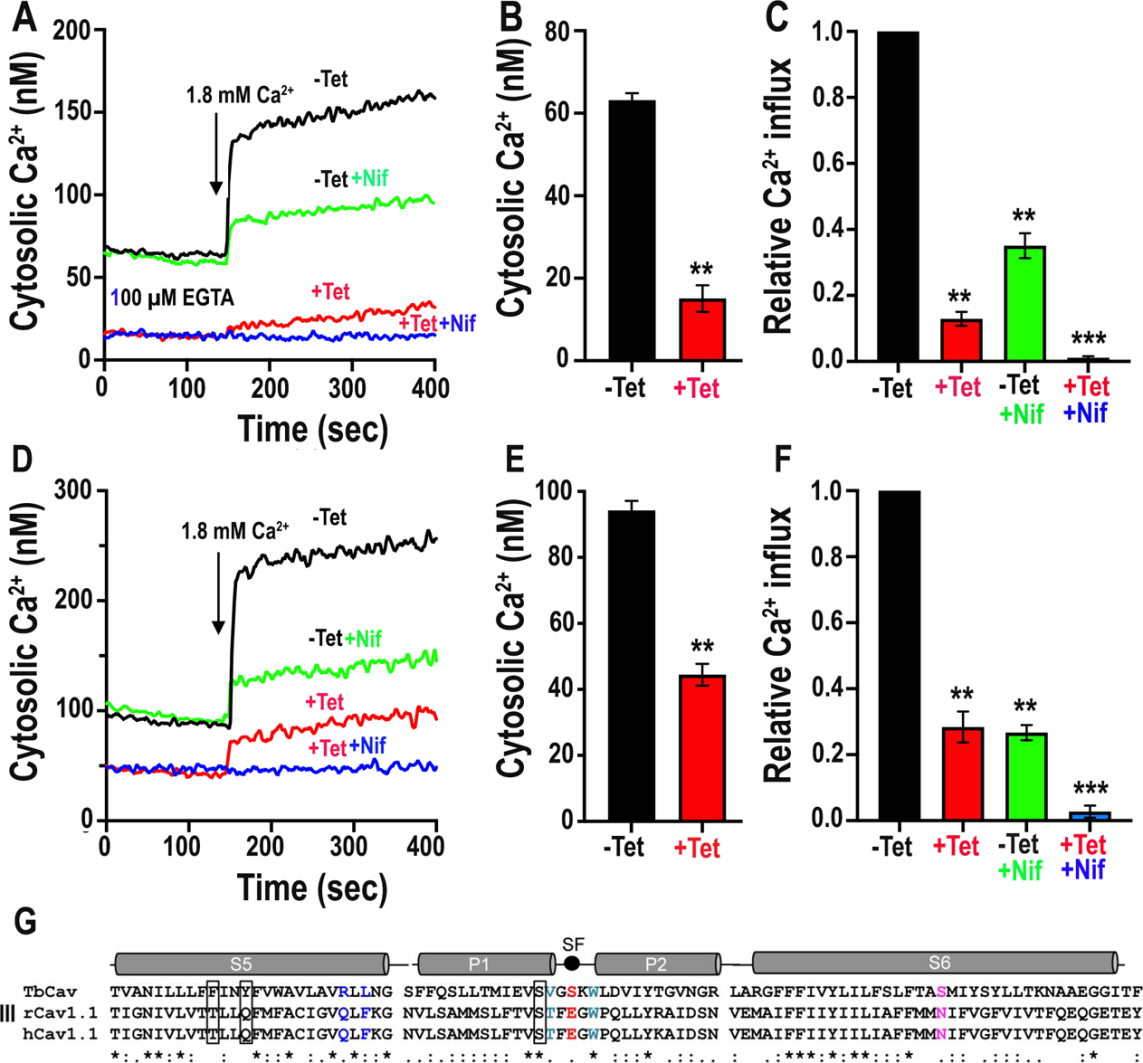
Ca^2+^ entry in BSF and PCF cells is inhibited by *TbCa_V_* RNAi and by nifedipine. (A) Addition of 1.8 mM Ca^2+^ increased the [Ca^2+^]_c_ of Fura 2/AM loaded BSF cells grown in the absence of tetracycline (black; −Tet), washed, and incubated with added calcium. When cells are grown in the presence of tetracycline (red; +Tet) there is a significant decrease in [Ca^2+^]_c_ (A,B) and an 87% decrease in Ca^2+^ entry (A,C). When control cells (−Tet) are incubated in the presence of nifedipine (green, +10 μM Nif), there is decrease in the effect of Ca^2+^ addition (A,C). When both conditions occur (+Tet, +10 μM Nif), there is practically no Ca^2+^ entry (blue) (A,C). Similar results were obtained with PCF cells grown for 4 days in the presence or absence of tetracycline (D–F). *P < 0.05; **P < 0.01: ***P < 0.001, *n* = 3, Student’s *t*-test. (G), sequence alignment of TbCa_V_, rabbit Ca_V_1.1 (rCa_V_1.1) and human Ca_V_1.1 (hCa_V_1.1) of the segments that participate in dihydropyridine (DHP) binding. Only one of the three more important amino acids (boxed in black) in the binding site (segment 5, S5 and pore domain 1, P1) is conserved in TbCa_V_. Other important amino acids in the selectivity filter (SF) and S6 region are also not conserved (in red, blue, and purple), as described in Figure S4.

We tested Ca^2+^ entry by adding 1.8 mM Ca^2+^ to suspensions of BSF cells preincubated in a buffer containing 100 μM EGTA (<50 nM extracellular Ca^2+^). Under these conditions, control parasites expressing a fully functional TbCa_V_ showed a cytosolic Ca^2+^ increase, which then plateaued. However, mutant parasites previously exposed to tetracycline for 2 days, and washed, had a lower steady-state cytosolic Ca^2+^ concentration ([Ca^2+^]_c_) (∼20 nM, as compared with ∼62 nM in the controls; Figure 6B) and were practically unable to increase cytosolic Ca^2+^ (87% reduction) suggesting that this is the main plasma membrane Ca^2+^ channel active in T. brucei BSF cells (Figure 6C). Interestingly, addition of the DHP nifedipine (10 μM), a mammalian Ca_V_ inhibitor, significantly reduced Ca^2+^ influx (Figure 6A,C) and addition of nifedipine to Tet + cells abolished Ca^2+^ entry, demonstrating the KD hypersensitivity to nifedipine. Similar results were obtained with PCF cells grown for 4 days in the presence and absence of tetracycline (Figure 6D–F). The lack of complete inhibition of Ca^2+^ entry by nifedipine could be due to differences in the binding site of DHPs to the Ca_V_ channels (Figure 6G). TbCa_V_ only conserves one of the three most important amino acids for DHP binding in rabbit and human Ca_V_1.1 (Figure 6G, boxed).^29^ These results suggest that more selective and specific inhibitors can be found.

In conclusion, TbCa_V_ is responsible for Ca^2+^ entry in T. brucei. There is strong evidence that TbCa_v_ is structurally and evolutionarily different from the animal homologues that can be exploited to develop specific and selective inhibitors. TbCa_V_ functions as a Ca^2+^ entry channel when expressed alone, as demonstrated by yeast complementation studies, by expression in HEK293T cells, and by the inhibition of Ca^2+^ entry into T. brucei by RNAi and nifedipine and is essential for the survival of T. brucei BSF and PCF cells. Therefore, we became interested in searching for new inhibitors. Without a good lead compound, we resorted to virtual screening, as described below.

### Construction of a Structural Model for TbCa_V_

We examined conformational ensembles of TbCa_V_ to perform docking analysis and thus virtual screening of inhibitors. Although the CryoEM structure of rabbit Ca_v_ (rCa_v_) is available,^29^ there is no experimental structure for TbCa_V_ or its close homologues. The low sequence identity between rCa_V_ and TbCa_V_ (<30%) renders it unfeasible to perform conventional template-based homology modeling. Instead, we used the denovo structure prediction method AlphaFold as it has been demonstrated to reach an accuracy level comparable to that of experiments.^30,31^ We first built a model for rCa_V_ de novo using AlphaFold, which is essentially the same as that obtained by CryoEM results, establishing a high degree of confidence for using the models built by AlphaFold. Then we went on to build a model for TbCa_v_ which exhibited overall similarity with that of the rCa_v_, based on CryoEM. However, at the drug binding sites, there are significant differences between TbCa_v_ and rCa_v_, suggesting the possibility of developing selective inhibitors. We used the cryo-EM structure for rCa_V_ (PDB: 6JP5) and the AlphaFold predictions forTbCa_V_, as working models.

We examined a series of known inhibitors of mammalian Ca_v_ (Table S1), which have high potency against mammalian Ca_v_. Such inhibitors showed much diminished or no activity against the T. cruzi version (TcCa_v_),^16,29,32^ suggesting the possibility to achieve selectivity in differentially inhibiting TbCa_V_. Further, in silico docking results showed poor binding of all these tested compounds in TbCa_v_ with many in the sampling population being outside of the binding pocket, consistent with the low potency from the experimental findings.

### Ensemble Molecular Docking Studies Using ZINC Database

The structures of TbCa_v_ and rCa_v_ were used to carry out microsecond-long molecular dynamics (MD) simulations. We performed all-atom MD simulations of TbCav and rCav in an explicit solvent and membrane environment. These simulations generated ensembles of conformations to further explore the differences between their binding sites. Notably, the dynamics of these sites differed significantly: The TbCa_v_ pocket was found to be somewhat smaller, more hydrophobic, and distinctly different in terms of the positions and orientations of the various side chain functional groups, suggesting excellent prospects for improving selectivity (Figure 7).

**Figure 7:**
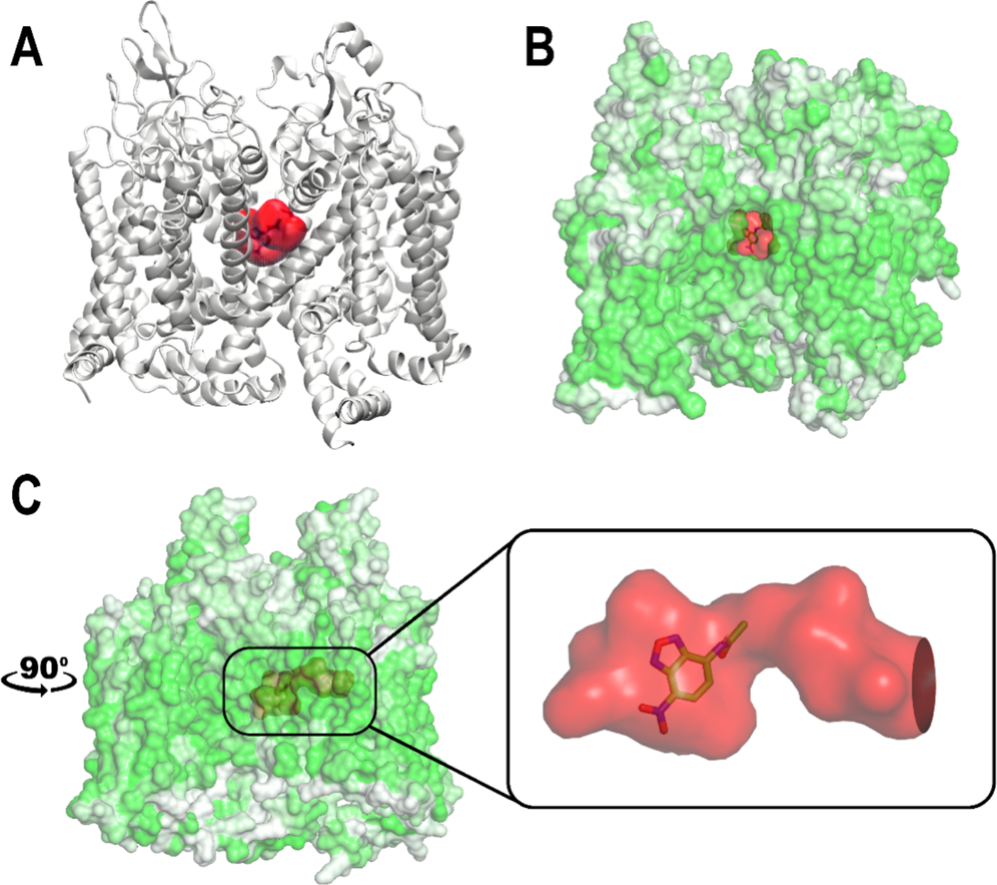
Location and shape of the cryptic binding pocket in TbCa_v_, identified through MD simulation. (A) Extracted frame of TbCa_V_ after principal component analysis (PCA) clustering, depicted in a ribbon representation with the cryptic pocket highlighted in red, showing its position within the protein’s secondary structure. (B) Surface representation of TbCa_V_, colored by hydrophobicity (greener = more hydrophobic, whiter = more hydrophilic), showing accessibility of tunnel-like pocket (red). (C) (Rotated 90 deg) The inset provides a close-up of the pocket in a surface view, showing the orientation of the docked ligand NBD-A within the pocket, with entry on the right.

We conducted ensemble molecular docking studies utilizing the ZINC database, a free database that provides over 35 million commercially purchasable compounds in ready-to-dock 3D formats. Employing ZINC, we docked approximately 12 million compounds into the binding sites of the TbCa_v_ and rCa_v_ ensembles of conformations using Autodock Vina^33^ and the ensemble docking approach.^34–37^ We identified the top 200 compounds that showed preferential binding to TbCav, based on stability, chemical reactivity, and avoidance of nondrug-like properties. We purchased 19 of these compounds and evaluated their efficacy in inhibiting T. brucei BSF proliferation. The most active compounds were two nitrobenzoxadiazole derivatives: *N*-(7-nitro-2,1,3-benzoxadiazol-4-yl) acetamide (or NBD-A) and 4-chloro-7-nitro-2,1,3-benzoxadiazole (or NBD-Cl), with EC_50_ values of 25 ± 3 and 62 ± 8 nM, respectively (Figure 8) (Table S2). In comparison, NBD-A and NBD-Cl cytotoxicity on Vero cells (which possess an L-type voltage-gated Ca^2+^ channel^38^) had EC_50_s values of 44.7 ± 3.6 μM and 28.2 ± 1.4 μM, respectively (1788 and 454 x EC_50_ for T. brucei, respectively). Figure 9 shows the inhibition of Ca^2+^ entry by NBD-A with an IC_50_ of 1.46 ± 0.03 μM. Figure 10 shows a docked pose of the ligand NBD-A in the binding pocket of the channel, highlighting key interactions. The identification of such a potent and selective inhibitor through virtual screening is very exciting, helps validate the computational approaches used, and lays a strong foundation for future optimization work.

**Figure 8:**
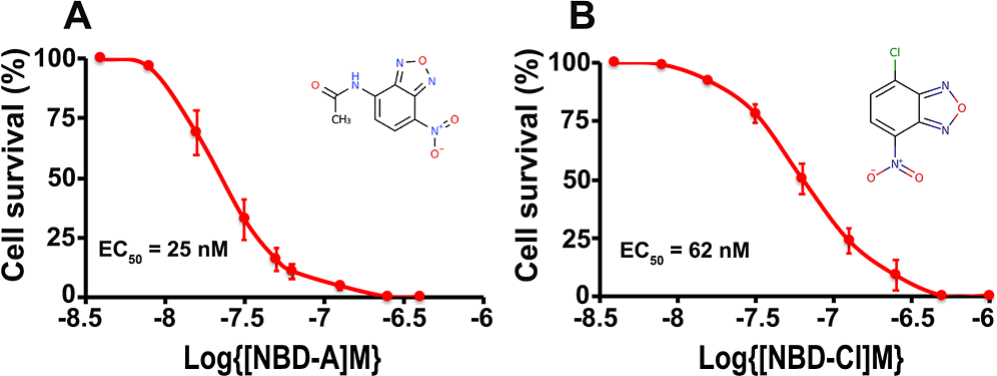
Effect of NBD-A and NBD-Cl on survival of T. brucei BSF. WT BSF (strain 427, 1 × 10^5^ cells/2 mL HMI-9 medium) were cultured at 37 °C for 2 days in the presence of different concentrations of NBD-A (A) or NBD-Cl (B) and live parasites were counted. The EC_50_s were calculated using GraphPad Prism software. Values are means ± s.d. of 3 experiments.

**Figure 9:**
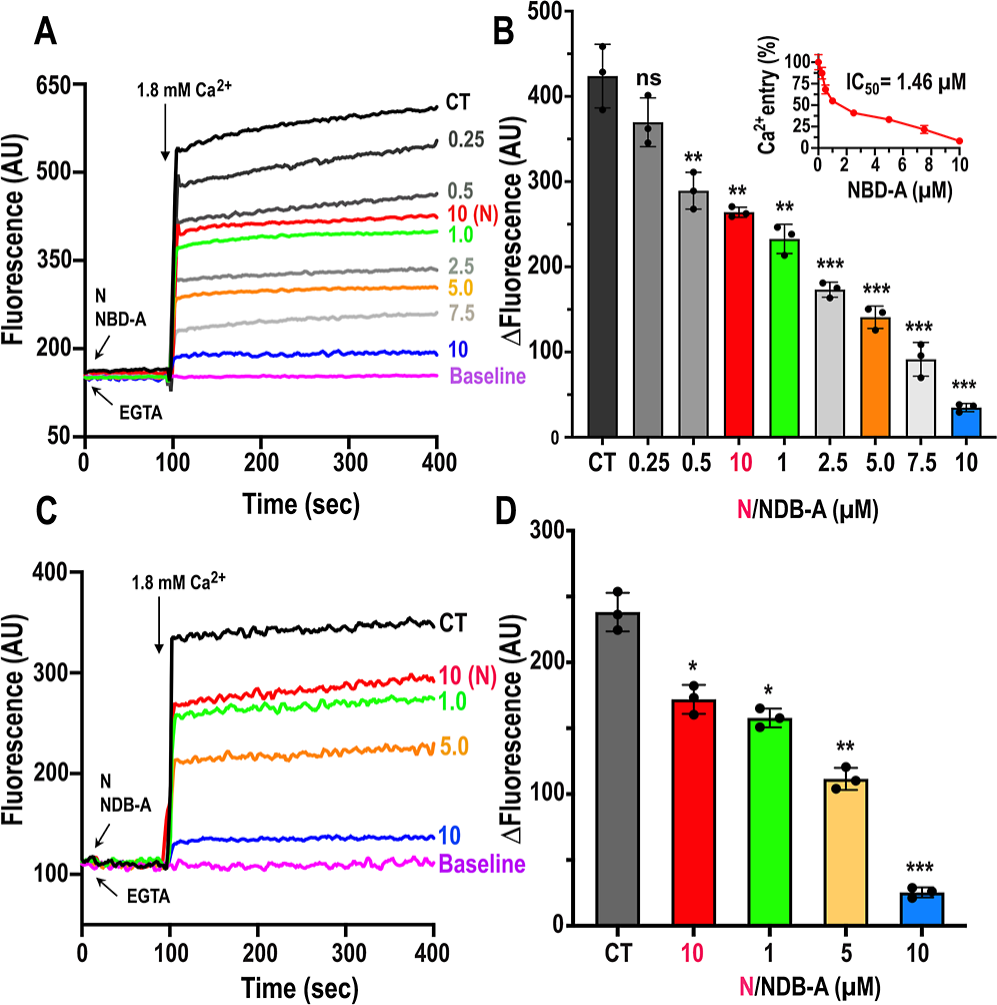
Ca^2+^ entry in BSF and PCF cells is inhibited by NBD-A and by nifedipine. Intracellular Ca^2+^ concentrations of 5 × 10^7^ wild-type BSF (A,B) and PCF (C,D) were measured by Cal590 signal fluorescence in arbitrary units of fluorescence (AU). An increase in Cal590 fluorescence indicates increasing cytosolic Ca^2+^. (A), 100 μM EGTA was initially added to remove extracellular Ca^2+^. Addition of 1.8 mM Ca^2+^ at 100s increased intracellular Ca^2+^ in Cal590-AM loaded BSF cells (CT, black). Addition of 10 μM Nif (red) or 0.5–10 μM NBD-A (grays, green, orange, and blue) showed a significant reduction of intracellular Ca^2+^ in the BSF cells. The EC_50_ were calculated using GraphPrism software, as shown (inset). Similar results were obtained with PCF cells (C,D). Values are means ± s.d. ns, no significant, *P < 0.05; **P < 0.01; ***P < 0.001, *n* = 3, Student’s *t*-test.

**Figure 10:**
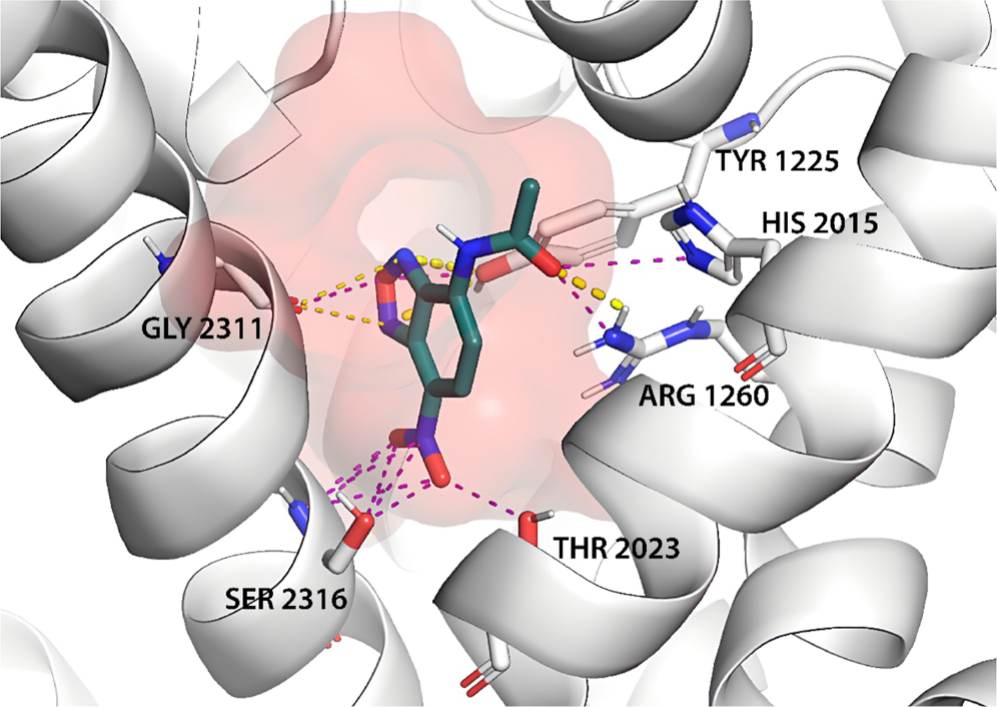
Docked pose of ligand NBD-A in the binding pocket of TbCa_V_, highlighting key interactions. Thick yellow dashed lines represent good polar contacts, thin yellow dashed lines indicate suboptimal polar contacts, and purple dashed lines represent potential electrostatic interactions.

## Discussion

We report the chemical and genetic target validation of the T. brucei Ca^2+^ entry channel and the ability to achieve selective inhibition of TbCa_V_. The channel localizes to the flagellar plasma membrane and is composed of a single subunit with low similarity to the α1 subunit of mammalian L-type Ca^2+^ channels. As occurs with mammalian cells, Ca^2+^ entry in T. brucei is sensitive to the Ca_V_ channel DHP inhibitor nifedipine, although at higher concentrations (high micromolar) than those needed for inhibiting the mammalian ortholog. Complementation studies in yeast and expression in HEK293T cells provided functional evidence of its role as a plasma membrane Ca^2+^ channel.

Trypanosomatids lack several types of plasma membrane Ca^2+^ channels but possess orthologs to TRP and Ca_v_ channels.^7^ We and others^39^ reported that T. brucei TRP channels (both TbTRPML and TbTRPP) exclusively localize to the lysosome whereas TbCa_V_ localized to the flagellar plasma membrane of T. brucei. Thus, far, TbCa_V_ is a sole Ca^2+^ channel identified for Ca^2+^ entry in the plasma membrane of T. brucei, which in contrast to T. cruzi and Leishmaniaspp., lacks Piezo channels.^40^

Ca_V_ channel inhibitors have been used for decades for the long-term treatment of hypertension and myocardial ischemia and are known to be among the most widely prescribed drugs worldwide because of their efficacy and few side effects.^41^ Similarly, selective inhibitors of TbCa_V_ offer great promise for treating T. brucei infection. Trypanosomes Ca_V_ channels have marked structural differences with mammalian homologues,^13^ suggesting the high probability of developing selective inhibitors with minimal effects on the host Ca_V_ channels as demonstrated with the new NBD derivatives that we have identified, which are potent and selective inhibitors of Ca^2+^ entry capable of killing T. brucei without significant cytotoxicity. Pharmacokinetic and pharmacodynamic issues of future drug candidates will need to be assessed to evaluate the efficacy and safety of any treatment.

NBD derivatives are electrophilic compounds that have been used as fluorescent probes for labeling biomolecules and as enzyme inhibitors. Some of these derivatives, like NBD-Cl, modify certain amino acids covalently and inhibit S. cerevisiae vacuolar H^+^-ATPase (V-ATPase).^42^ NBD-Cl was shown to attack a tyrosine residue in or near the catalytic site of the vacuolar membrane V-ATPase subunit A leading to its rapid inactivation.^42,43^ About 1 μM NBD-Cl caused half maximal V-ATPase inactivation at neutral pH.^43^ NBD-Cl (at 0.1 mM) was also able to inhibit the bovine F_1_-ATPase by covalent reaction with Tyr311 in one of the three catalytic β subunits of the complex.^44^ The NBD derivative 6-(7-nitro-2,1,3-benzoxadiazol-4-ylthio)­hexanol (NBDHEX) is a strong inhibitor of glutathione S-transferases and accumulates in tumor cells avoiding the extrusion mechanism mediated by the multidrug resistance protein pumps and has been proposed as a potential anticancer drug.^45^

In conclusion, our docking and virtual screening results validated our computational approach as a powerful tool in helping identify potent and selective TbCa_V_ inhibitors de novo.

## Methods

### Culture Methods

Cultivation of procyclic (PCF) and bloodstream form (BSF) of T. brucei Lister strain 427 was carried out as described previously.^46^ PCF 29-13 (*T7RNAP NEO TETR HYG*) coexpressing T7 RNA polymerase and Tet repressor was a gift from George A. M. Cross (Rockefeller University, New York, NY) and was grown in SDM-79 medium^47^ supplemented with hemin (7.5 μg/mL) and 10% (v/v) heat-inactivated (HI) FBS and at 27 °C in the presence of G418 (15 μg/mL) and hygromycin (50 μg/mL) to maintain the integrated genes for T7 RNA polymerase and tetracycline repressor, respectively.^48^ BSF (single marker-SM strain) was also a gift from George A. M. Cross and was grown at 37 °C in HMI-9 medium^49^ supplemented with 10%(v/v) FBS, 10% (v/v) serum plus (JRH Biosciences), and 2.5 μg/mL G418.

HEK293T cells were cultured in High Glucose Dulbecco’s Modified Eagles Media (HG-DMEM) supplemented with 10% HI-FBS, 100 IU/ml penicillin, 100 μg/mL streptomycin, and incubated at 37 °C with 5% CO_2_. Vero cells were grown and maintained at 37 °C with 5% CO_2_ using RPMI-1640 media supplemented with 10% heat-inactivated newborn calf serum, 25 μg/mL gentamycin, 100 IU/ml penicillin, 100 μg/mL streptomycin, and 2 mM l-glutamine.

### Cloning of the Voltage-Gated Ca^2+^ Channel

To clone and express the voltage-gated Ca^2+^ channel (*TbCa_v_*), the full-length cDNA of TbCa_v_ (8079 bp) was amplified with Q5 High-Fidelity DNA polymerase (NEB) from T. brucei genomic DNA by long PCR using the primers TbCa_v_-HF and TbCa_v_HA-HR, TbCa_v_-YF and TbCa_v_HA-YR listed in Table S3, and then cloned into *Nhe*I-*Xho*I-cut pcDNA3.1 mammalian expression vector (Thermo Fisher Scientific) or *Hin*dIII-*Bam*HI-cut pYES2 yeast expression vector (Thermo Fisher Scientific) using the In-Fusion Cloning Kit (Clontech) to generate pcDNA3.1-*TbCa_v_-HA* and pYES2-*TbCa_v_-HA*, respectively. The double-stranded nucleotide sequences of HA-tagged TbCa_v_ in expression vector pcDNA3.1 or pYES2 were confirmed by sequencing at Azenta.

### Expression of *TbCa_v_* in HEK Cells

Log-phase HEK293T cells were seeded on half-volume 6-well plates (5 × 10^5^ cells/well) and incubated overnight to be 50%–60% confluent. HEK293T cells were transiently transfected with pcDNA3.1-*TbCav-HA* (1.2 μg DNA/well) using GeneJuice (Novagen), according to the manufacturer’s instructions. Two days after transfection, gene expression in HEK cells was detected by Western blot analyses. The transfected HEK cells were used for Ca^2+^ measurements.

### Yeast Complementation

Yeast complementation was performed as described.^25^ Briefly, the *cch1/mid1* mutant^24^ was transformed via LiOAc-mediated method^50^ with either (pYES2-*TbCa_v_-HA*) or the empty vector pYES2 and transformants selected on yeast minimal medium without uracil. To test for complementation, sterile cellulose filter discs (6 mm diameter and 45 μm pore size) were soaked with 10 μg of synthetic alpha factor (Sigma T6901) and placed on nascent lawns of the transformed *cch1/mid1* mutants or WT strain W303a (*MATa can1-100 ade2-1 his3-11,15 leu2-3 trp1-1 ura3-1*) and pictures taken after 48 h of growth at 30 °C. The assay was repeated 3 times.

### *TbCa_v_* RNAi Constructs

To knockdown the expression of the *TbCa_v_* (XM_817448.1, Tb427.10.2880) by double-stranded RNA expression, the inducible T7 RNA polymerase-based protein expression system and the p2T7^Ti^ vector with dual-inducible T7 promoters were employed. A 501-bp cDNA fragment of TbCa_v_ targeted to the nucleotides 2993–3494 of the open reading frames (ORFs) was amplified using the primers TbCa_v_-IF and TbCa_v_-IR listed in Table S3, digested with *Bam*HI and *Hin*dIII, and cloned into p2T7^Ti^ vector. The recombinant construct p2T7­(*TbCa_v_*) was confirmed by sequencing at Azenta, NotI-linearized, and purified with Zymo’s DNA Clean and Concentrator for cell transfections. TbCa_v_ have novel nucleotide sequences without homologues (>20 nucleotide identity) in T. brucei genome/transcript databases (TriTrypDB), suggesting the absence of any other potential gene targets.

### Generation of “Spaghetti Monster” Fluorescent Protein Tagging Cassettes

The one-step epitope-tagging protocol reported by Oberholzer et al.^20^ was used to produce C-terminal smFP-epitope tagging cassettes of TbCa_v_ and TbTRPP (TriTrypDB gene ID numbers Tb427.10.2880 and Tb427.08.850, respectively) for PCR-mediated transfection of T. brucei PCF and BSF trypanosomes. In brief, the PCR forward and reverse primers included terminal 144 nucleotides of each ORF before its stop codon and the reverse complement of the first 141 nucleotides of the 3′UTR, respectively, followed in frame by the 21–26 nucleotides of the backbone sequences of pMOTag2 mV vector.^51^ The smV5-tagging cassettes containing smFP with multiple V5 epitope tags (smV5) and a puromycin resistant gene as the selection marker were generated for cell transfection by PCR using pMOTag2 mV^51^ as template with the corresponding PCR primers of the TbCa_v_ or *TbTRPP* gene (Table S3).

### T. brucei Cell Transfection

Mid log phase PCF trypanosomes (∼5 × 10^6^ cells/ml) were harvested by centrifugation at 1000*g* for 7 min, washed with Cytomix buffer (2 mM EGTA, 3 mM MgCl_2_, 120 mM KCl, 0.5% glucose, 0.15 mM CaCl_2_, 0.1 mg/mL BSA, 10 mM K_2_HPO_4_/KH_2_PO_4_, 1 mM hypoxanthine, 25 mM Hepes, pH 7.6) and resuspended in 0.45 mL of the same buffer at a cell density of 2.5 × 10^7^ cells/ml. The washed cells were mixed with 50 μL of NotI-linearized plasmid DNA or purified PCR products (10 μg) in a 0.4 cm electroporation cuvette and subjected to two pulses from a Bio-Rad Gene Pulser electroporator set at 1.5 kV and 25 μF. The stable transformants were obtained in SDM-79 medium supplemented with 15% FBS plus appropriate antibiotics (15 μg/mL G418, 50 μg/mL hygromycin, 5 μg/mL phleomycin, or/and 2 μg/mL puromycin).

For the BSF, 10 μg of NotI-linearized plasmid DNA or purified PCR products (<10 μL) were used per 4 × 10^7^ mid log phase cells in 100 μL AMAXA Human T-cell Nucleofector solution. Electroporation was performed using 2 mm gap cuvettes with program X-001 of the AMAXA Nucleofector. Following each transfection, stable transformants were selected and cloned by limiting dilution in HMI-9 medium containing 15% FBS with appropriate antibiotics (2.5 μg/mL G418, 2.5 μg/mL phleomycin, or/and 0.1 μg/mL puromycin) in 24-well plates.

Antibiotic-resistant cell lines or clones were further characterized, as described below. RNAi was induced with 1 μg/mL fresh tetracycline when the cells were at a density of 2 × 10^6^ PCF or 1 × 10^5^ BSF cells/ml. The cells were counted using a hemocytometer, and growth curves were generated for clones or cell lines over a period of 8 days for PCF and 4 days for BSF. The correct smV5-tagging of TbCa_v_ or TbTRPP was confirmed by PCR followed by sequencing and Western blot analyses.

### Immunofluorescence Microscopy

T. brucei cells were harvested by centrifugation. BSF trypanosomes were washed in ice-cold PBS with 1% glucose and fixed with 1% paraformaldehyde in PBS at 4 °C for 1 h. PCF trypanosomes were washed with PBS and then fixed with 4% paraformaldehyde in PBS at room temperature for 1 h. The fixed parasites were washed twice with PBS, allowed to adhere to poly-l-lysine-coated coverslips, and permeabilized with 0.3% Triton X-100/PBS for 3 min for PCF or 0.1% Triton X-100/PBS for 5 min for BSF. After blocking with PBS containing 3% BSA, 1% fish gelatin, 50 mM NH_4_Cl and 5% goat serum for 1 h, trypanosomes were stained in 3% BSA/PBS with the purified HA.11 clone 16B12 monoclonal antibody against HA (1:50), mouse monoclonal antibody against FCaBP (1:10), mouse monoclonal antibody against V5 (1:100), mouse anti-p67 monoclonal antibody (1:200), mouse monoclonal antibody against tubulin (1:1000), rabbit monoclonal antibody against V5 (1:500) for 1 h. After thoroughly washing with PBS containing 3% BSA, cells were incubated with Alexa 488-conjugated goat antimouse or antirabbit antibodies and Alexa 546-conjugated goat antirabbit or antimouse antibodies at 1:1000 for 1 h. Immunofluorescence of yeast was performed as described previously.^51^

After being labeled with primary and secondary antibodies, the trypanosome or yeast cells on the coverslips were counterstained with 4′,6-diamidino-2-phenylindole (DAPI) before mounting with Gold ProLong Gold antifade reagent (Molecular Probes). Differential interference contrast and fluorescent optical images were captured using an Olympus IX-71 inverted fluorescence microscope with a Photometrix CoolSnap^HQ^ charge-coupled device camera driven by DeltaVision software (Applied Precision, Seattle, WA). Images were deconvolved for 15 cycles using SoftwarX deconvolution software. Pearson’s correlation coefficients were calculated using the SoftwarX software by measuring the whole-cell images.

### Western Blot Analyses

The T. brucei cells were harvested and washed twice in buffer A with glucose (BAG) which contained 116 mM NaCl, 5.4 mM KCl, 0.8 mM MgSO_4_, 5.5 mM d-glucose, and 50 mM HEPES at pH7.0. The cells were lysed with RIPA buffer I (150 mM NaCl, 20 mM Tris/HCl, pH 7.5, 1 mM EDTA, 1% SDS, and 0.1% Triton X-100) containing protease inhibitor tablet in ice for 1 h. The yeast cells were digested with zymolyase and then lysed in RIPA buffer I as described previously.^51^ Transfected HEK cells were detached with Trypsin–EDTA solution (Thermo Fisher Scientific) and rinsed twice with BAG. The HEK cells were lysed in RIPA buffer II (150 mM NaCl, 50 mM Tris/HCl, pH 8.0, 0.1% SDS, 0.5% sodium deoxycholate, and 1% Triton X-100) containing a protease inhibitor tablet in ice for 1 h. The protein concentration was determined by using a Pierce BCA protein assay kit with the microplate reader.

Total cell lysates were mixed with 2 × Laemmli sample buffer (BioRad) at 1:1 ratio (volume/volume), directly loaded (for T. brucei and HEK proteins), or loaded after boiling for 5 min (for yeast proteins). The separated proteins were transferred onto nitrocellulose membranes using a Bio-Rad transblot apparatus. The membranes were blocked with 10% nonfat milk in PBS-T at 4 °C overnight. The blots were incubated with mouse antibodies against V5 (1:2500), mouse antibodies against HA (1:1000), mouse antibodies against tubulin (1:10,000), or rabbit antibodies against hexokinase (1:20,000) for 1 h. After five washings with PBS-T, the blots were incubated with horseradish peroxidase conjugated antimouse or antirabbit IgG (H + L) antibody at a dilution of 1:15,000 for 1 h. After washing five times with PBS-T, the immunoblots were visualized using Pierce ECL Western blotting substrate (Thermo Fisher Scientific), according to the manufacturer’s instructions.

### In Vitro BSF Drug Sensitivity Assays

Mid log phase wild-type BSF (strain 427) were centrifuged and resuspended to a final density of 1 × 10^7^ cells per milliliter in HMI-9 medium. Our screening was done first using a concentration of each compound of 1 and 10 μM. If the compounds showed no inhibition of T. brucei proliferation at those concentrations, we did not further test them and indicated EC_50_ > 10 μM for these compounds. Only those that were active in the initial screening were tested at different concentrations to obtain the EC_50_ values, since the aim was to find an effective trypanocidal compound of those selected by docking studies. The BSF trypanosomes were incubated with a range of inhibitor concentrations (0.01–10 μM) and without inhibitor as control in 12-well plates (1 × 10^5^ cells/2 mL HMI-9 medium/well) at 37 °C for 2 days. The viable cells were counted using a hemocytometer under a microscope. Each treatment was triplicated. The EC_50_s were calculated using GraphPad Prism software. The structures of all of the compounds tested are shown in Table S2. The purity determination of the top inhibitors was provided by the commercial vendor and it was 90% for NBD-A and 98% for NBD-Cl. Compounds 1–10 were provided by Ambinter, compounds 11–17 by MolPort, and compounds 18–19 by AK Scientific.

### Cytotoxicity of Drug Compounds on Vero Cells

The cytotoxicity was tested using AlamarBlue assay as described previously,^52^ with some modifications. In brief, confluent monolayers of Vero cells were seeded in 96-well plates (black, clear bottom) in 150 μL of RPMI-1640 with 10% HI-FBS. After overnight incubation, wells were washed once with the fresh medium to eliminate any detached host cells, and drug compounds were added in serial dilutions and mixed well in RPMI media in 150 μL volumes. Each dilution was tested in quadruplicate. Each plate also included controls with host cells only. Plates containing drug dilutions were incubated at 37 °C and 5% CO_2_ for 96 h. After 4 days, AlamarBlue (15 μL) (Bio-Rad) was aseptically added to the cultures and incubated at 37 °C for 6 h. After incubation, absorbance was measured at 570 and 600 nm on a spectrophotometer. The percent difference in reduction between treated and control cells in cytotoxicity was calculated by the formula as described in.^52^

### Ca^2+^ Measurements in T. brucei Cells

The harvested T. brucei cells were washed twice with BAG and loaded in BAG with 5 μM Fura-2/AM (Molecular Probes) or 10 μM Cal-590/AM (AAT Bioquest) plus 1.5% sucrose for 30 min in a 30 °C water bath with mild agitation and rinsed twice by centrifugation to remove extracellular dye. The cells were resuspended to a final density of 1 × 10^9^ cells per ml in BAG. For fluorescence measurements, a 50 μL-aliquot (5 × 10^7^ cells) of the cell suspension was added to 2.45 ml BAG containing 100 μM EGTA (<10 μM extracellular Ca^2+^) in a cuvette. Ca^2+^ entry was monitored by adding 1.8 mM Ca^2+^ to T. brucei suspension to the cuvette on a Hitachi F7000 spectrofluorometer with excitation wavelengths of 340/380 nm and emission wavelengths of 510 nm for Fura-2. Excitation wavelength at 540 nm and emission wavelength at 590 nm were used for Cal-590, which were unaffected by the fluorescence of NBD-A (excitation: 400 nm, emission: 420 nm). A Hitachi F7000 spectrofluorometer was used, as previously described.^53^ Ca_v_ inhibitors (nifedipine, NBD-A) were added as indicated in the Figures.

### Ca^2+^ Measurements in HEK Cells

Ca^2+^ measurements were performed 2 days after the transfection of HEK293T cells. Transfected and untransfected HEK cells were detached with Trypsin–EDTA solution (Thermo Fisher Scientific), resuspended in HG-DMEM, and then rinsed twice with BAG. The HEK cells were loaded in BAG with 5 μM Fura-2/AM. Ca^2+^ entry was monitored by adding 0.6 mM Ca^2+^ to HEK suspension the cuvette on a Hitachi F7000 spectrofluorometer, as described above.

### Molecular Dynamics Simulation

Since an experimental structure for the T. brucei voltage-gated Ca^2+^ channel was not available, a predicted model for the T. brucei (Uniprot ID: Q38BV7)^54^ generated by AlphaFold was chosen for the study. The segments within intracellular regions were disordered and predicted with low confidence. Most of these regions were removed, while segments in the transmembrane and extracellular regions were preserved for the simulation. For the simulation of mammalian Ca^2+^ channel, the cryo-EM structure of rabbit L-type Ca^2+^ channel subunit alpha-1S was used (PDB: 6JP5)^29^ which shares 93% sequence identity to human L-type Ca^2+^ channel (Uniprot ID: Q13698).^54^ Some missing regions (residues 145–160, 856–865, 884–891, and 1207–1231) of the experimental structure were also modeled by AlphaFold. There are a total of 2693 residues in the parasitic channel and 1873 residues in the mammalian channel, but the sections used for the simulation of the parasitic channel were 317–636, 1031–1288, and 1759–2354, while for the mammalian channel, they were 32–376, 418–687, and 788–1506. Several studies had reported a connection between the DHP binding site and calcium coordination to the selectivity filter in the case of mammalian channel.^55–57^ The binding of Ca^2+^ to the selectivity filter has been found to be essential for effective DHP binding and channel closure.^58^ Therefore, Ca^2+^ was added to the parasitic protein model in the region of the selectivity filter. The open N-termini were capped by an acetyl group and the C-termini were methyl amidated to neutralize the free ends.

The membrane was prepared using CHARMM-GUI, a web-based tool for setting up complex biomolecular systems.^59^ Three classes of glycerophospholipids: phosphatidylcholine (PC) and phosphatidylethanolamine and sphingomyelin together constitute more than 80% of the parasite membrane phospholipids.^60–63^ Same classes of phospholipids are also present in skeletal muscle membrane but in a different ratio of subtypes according to variations in fatty acid chain and their saturation.^64,65^ The final choice of phospholipids for the MD simulation was based on the concentrations of these three phospholipids in the parasite’s membrane and skeletal muscle membrane, as well as the availability of parameters in the selected lipid force field.^66^ The lipids were placed in a ratio resembling the native lipid composition using a replacement method, ensuring equal area coverage in the upper and lower leaflets. The protonation states of all residues were calculated at pH 7, and the system was neutralized by adding Na^+^/Cl^–^ ions after lipid insertion. Isotonic conditions were achieved by supplementing the Na^+^/Cl^–^ ions. Subsequently, the residues of the prepared system were renamed to make it compatible with the AMBER MD program.^67^

The AMBER ff19SB force field parameters^68^ were used for the protein, while lipid21 parameters^66^ were employed for the membrane lipids. TIP3P solvent model was used to solvate the system. A restrained minimization was performed using steepest descent for first 2000 steps followed by conjugate gradient for 3000 steps to optimize water interactions gradually relaxing the restraints on protein and lipids from 500 kcal/mol·Å^2^ to 0 kcal/mol·Å^2^. The systems were gradually heated from 100 to 300 K using five rounds of simulations totaling 2.5 ns under *NVT* conditions, employing a Langevin thermostat with a collision frequency of 1 ps^–1^. During heating, positional harmonic restraints were applied to both the membrane and protein, with the strength of the restraints gradually reducing from 500 kcal/mol·Å^2^ for the initial stage, followed by reductions to 300, 100, 50, and finally 10 kcal/mol·Å^2^. Subsequently, the system was equilibrated at constant pressure (NPT) for 5 ns with backbone restraints of 10 kcal/mol·Å^2^. SHAKE was employed to constrain bonds with hydrogen to enable the use of a 2 fs time step. Long-range interactions were truncated at 9 Å using the particle mesh Ewald method.^69^ A Monte Carlo barostat with a coupling constant of 1 ps was used to regulate the pressure to 1 bar. The production run was conducted without any restraints under the same conditions for a duration of 1.2 μs. The initial 200 ns of production run were excluded from analysis. Protein coordinates were saved every 1 ps, resulting in 1 million frames from each simulation for analysis.

### Principal Component Analysis

The frames generated by MD simulation were clustered by using PCA on the active site region of the calcium channels. A graph of the top principal components (PC1 and PC2) against each other allows visualization of clusters or basins corresponding to different conformational states of the protein. A representative conformation was selected from each cluster to perform docking.

### Virtual Screening

The virtual screening was performed on a library of in-stock drug-like molecules from the ZINC20 database^70^ using Vina-GPU, a GPU version of AutoDock Vina.^71^ The obtained representative frames were extracted from the respective MD trajectories in the form of PDB files. Nonpolar hydrogen atoms were merged, and Kollman charges were added to each protein file using MGLtools 1.5.7.^72^ The prepared protein structures were saved in a PDBQT format. The used ligands were preprepared in PDBQT format, with partial charges assigned using ASMOL 7.1 through CM2 (charge model 2) mapping of Mulliken charges.^73^ The grid center coordinates were chosen by taking the centroid of all of the active site residues. A grid size of 24 Å × 24 Å × 24 Å was chosen for the docking as it effectively covers all of the binding site region.

The ligands were ranked based on their binding scores, prioritizing those that exhibit a higher affinity for T. brucei’s channel compared to their binding affinity for the mammalian counterpart.

### Statistical Analyses

All values are expressed as means ± standard deviations. Significant differences between treatments were compared by using an unpaired Student *t*-test. Differences were considered statistically significant at a *P* of < 0.05, and *n* refers to the number of experiments performed. All statistical analyses were conducted using GraphPad Prism 5 (GraphPad Software, San Diego, CA).

## Supplementary Material



## Notes

Phylogenetic tree of Ca_V_ channels; flagellar localization of tagged TbCa_V_ in procyclic cells; IFA of RNAi cells; topological structure and sequence alignment of Ca_V_ channels; known vertebrate calcium channel inhibitors; effect of compounds on T. brucei survival; and primers used in the study (PDF)
