# Supplementary material for: Chemical and Genetic Validation of an Essential Calcium Entry Channel of Trypanosoma brucei as a Therapeutic Target
Source: ACS Infect Dis. 2025 Jun 3;11(6):1741–52. doi: 10.1021/acsinfecdis.5c00329 (PMC12172032; doi:10.1021/acsinfecdis.5c00329)
Supplement: Supplementary file 1 [file id5c00329_si_001.pdf]

Supporting Information

Chemical and Genetic Validation of an Essential Calcium Entry Channel of *Trypanosoma brucei* as a Therapeutic Target

Guozhong Huang[1], Harmanpreet Singh[2], Priti Singh[2], Rohit Kumar Varshnaya[2], Donald Hamelberg[2], Binghe Wang[2] and Roberto Docampo[1,*]

[1]Center for Tropical and Emerging Global Diseases and Department of Cellular Biology, University of Georgia, Athens, GA 30602, United States

[2]Department of Chemistry, Georgia State University, Atlanta, GA 30303, United States

[*]Adress correspondence to Roberto Docampo, rdocampo@uga.edu

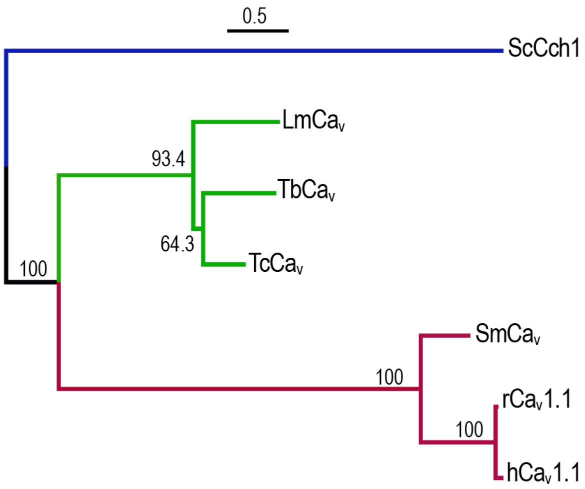

Figure S1. Phylogenetic tree of Ca_v channels in yeast, pathogenic parasites and mammals. The TriTrypDB and GeneBank accession numbers for seven distinct Ca_v channels from diverse organisms are as follows: CAA97244.1 (*Saccharomyces cerevisiae* CCH1, ScCch1), LmjF.34.0480 (*Leishmania major* Ca_v, LmCa_v), Tb427.08.850 (*Trypanosoma brucei* Ca_v, TbCa_v), TcYC6_0112380 (*Trypanosoma cruzi* Ca_v, TcCa_v), XP_002578175.1 (*Schistosoma mansoni* Ca_v, SmCa_v), P07293 (rabbit Ca_v1.1, rCa_v1.1), and Q13698 (human Ca_v1.1, hCa_v1.1), respectively. Protein sequences from the selective species were iteratively aligned with MUSCLE (https://www.genome.jp/tools-bin/clustalw) [1] and used to construct a phylogenetic tree with 100 bootstrap replicates using PhyML 3.0 [2]. Branch length scale bar corresponds to a protein distance of 50 changes per 100 amino acid positions, and branch support values quantify the confidence, as shown.

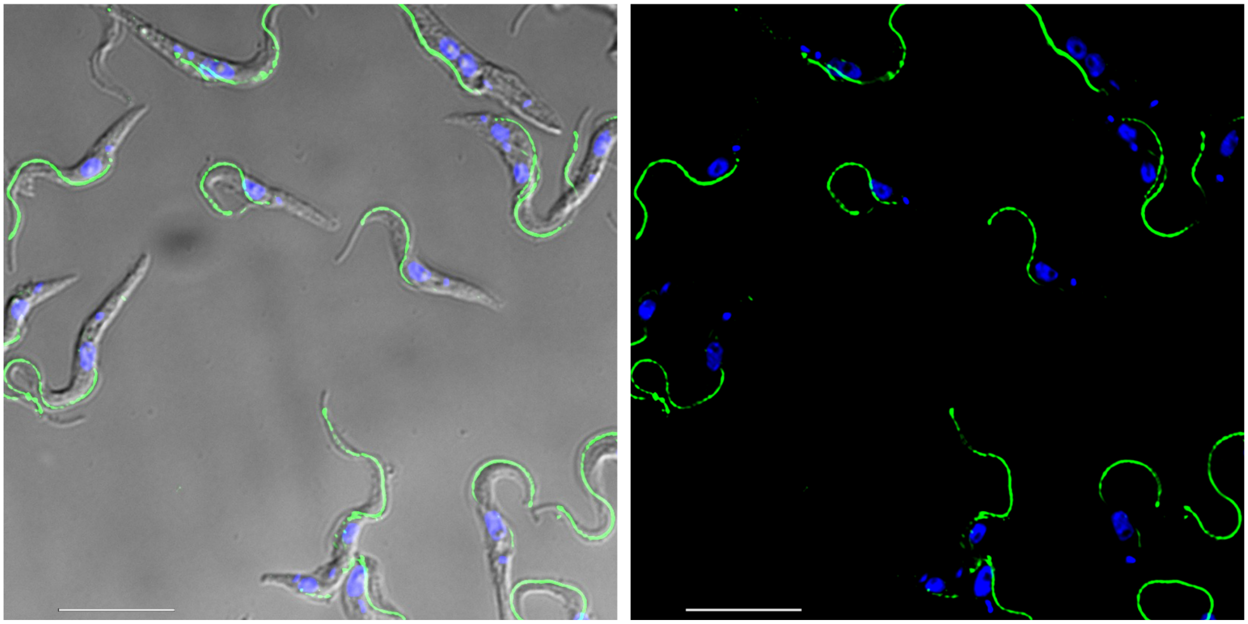

Figure S2. Flagellar localization of smV5-tagged TbCa$_V$ in PCF cells. IFA showing the flagellar localization of TbCa$_V$-smV5, and the absence of the fluorescence signal near the tip of the flagellum. Scale bars = 10 µm.

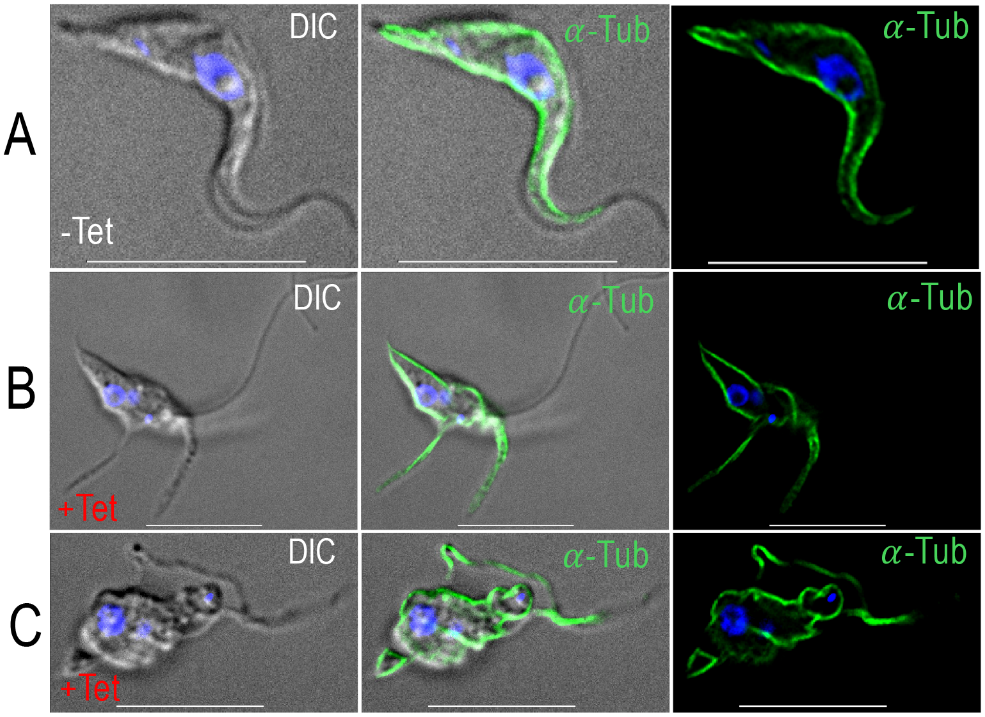

Figure S3. IFA of *T. brucei* PCF *TbCa$_v$*-RNAi cells grown in the absence (Tet-) or presence (Tet+) of 1 µg/ml tetracycline for 20 hours. Cells are stained with a-tubulin and DAPI. RNAi cells are multinucleated, multiflagellated and have flagellar detachment from the cell body. Left images are DIC. Scale bars = 10 µm.

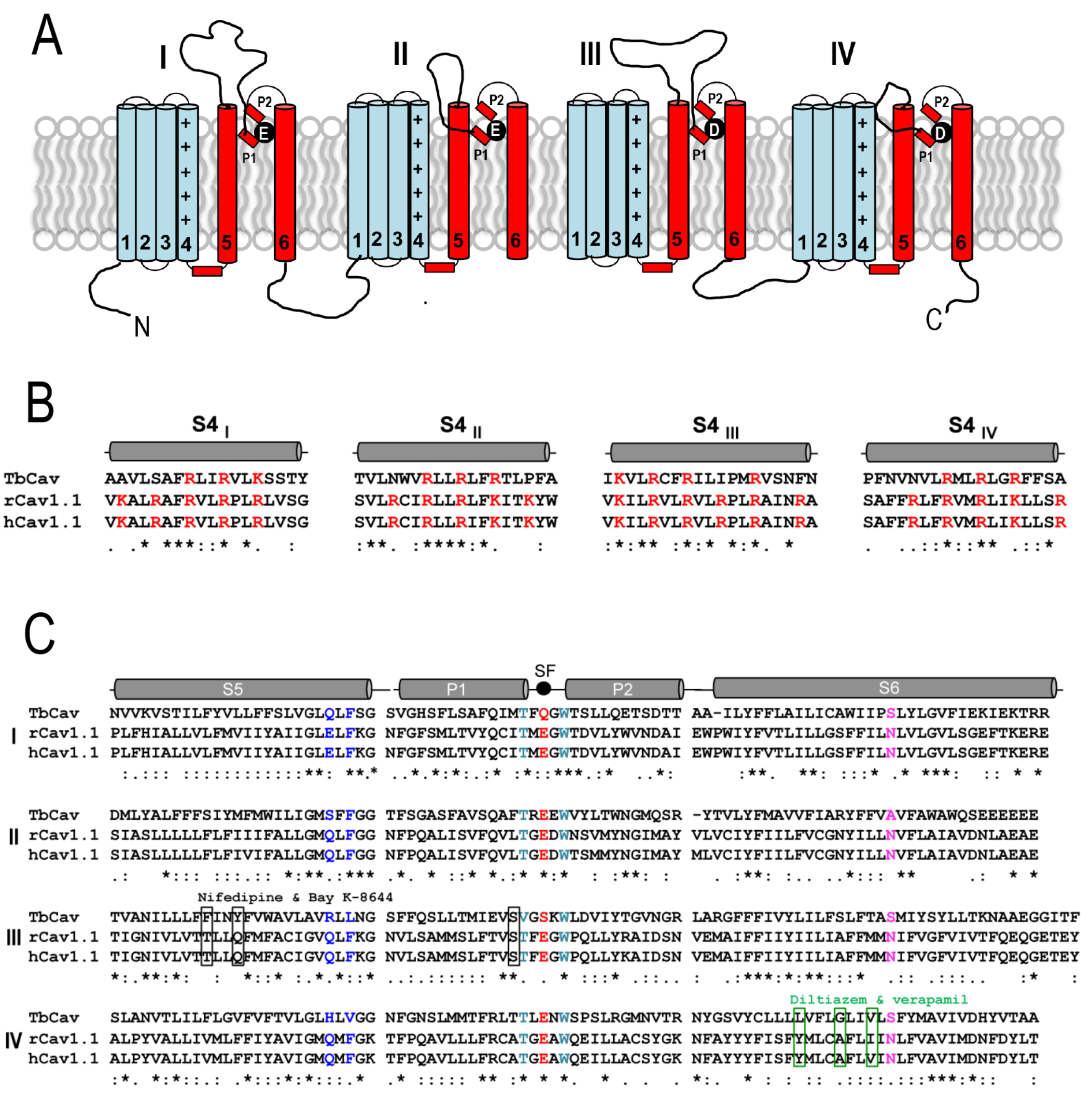

Figure S4. Topological structure and sequence alignment of Ca_v channels. A, The general topology of eukaryotic voltage-gated Ca^{2+} channel (Ca_v). Ca_v composes four repeats (I, II, III, IV). Each repeat is composed of six transmembrane helical segments, S1–S6. $S4_{I-IV}$ helices in each repeat form the voltage sensor domain (VSD) with five positive charge (+) residues, and the S5 and S6 helices, together with their intervening sequences, from the four repeats enclose two pore domains (P1 & P2) and a Ca^{2+} selective filter (SF) with the critical acidic residues (E, D). B, Sequence alignment of the voltage sensor domains ($S4_I$, $S4_{II}$, $S4_{III}$, and $S4_{IV}$) of *T. brucei* Ca_v (TbCa$_v$) with rabbit Ca_v1.1 (rCa$_v$1.1) and human Ca_v 1.1 (hCa$_v$1.1). The gating basic or positive charge residues (R, K) are indicated in red. C, Sequence alignment of the pore domains (S5-P1-SF-P2-S6 region) of TbCa$_v$ with rCa$_v$1.1 and hCa$_v$1.1. The QxF motif highly conserved in the S5 segments of mammalian Ca_v channels (mCa$_v$) is shown in blue. The critical residue (E) important for Ca^{2+} influx and the invariant residues (T and W) in the SF of mCa$_v$ channels are highlighted in red and in light blue, respectively. The critical residues participated in the binding of nifedipine & Bay K-8644, or diltiazem & verapamil, which were identified by the previous Cryo-EM studies on mCa$_v$ channels, are boxed in black and in green, respectively.

Table S1. Known vertebrate calcium channel inhibitors

**Inhibitor**	**Vertebrate Ca_V IC_{50} (nM)**	**Growth Inhibition (*T. cruzi*)**
Nifedipine	100 (rat) 200 (frog) [3, 4]	22.6 µM (IC_{50})[5]
Nicardipine	60 (human)[6]	40% at 10 µM[7]
Felodipine	0.15 (porcine)[8]	32% at 10 µM[7]
Lacidipine	82 (human)[9]	30 µM (IC_{50})[10]
Isradipine	4.2 (rat)[11]	20 µM (IC_{50})[10]
Amlodipine	3.0 (rabbit)[12]	8 µM (IC_{50})[10]
Nitrendipine	1.0 (rabbit)[13]	27 µM (IC_{50})[10]
Verapamil	65 (porcine)[8]	No activity at 100 µM[10, 14]
Diltiazem	260 (porcine)[8]	No activity at 100 µM[10, 14]

Table S2. Effect of nineteen compounds on survival of *T. brucei* BSF

EC_{50}	Zinc ID	Structure	Name	Smiles
>10 µM<br>1	ZINC4622727	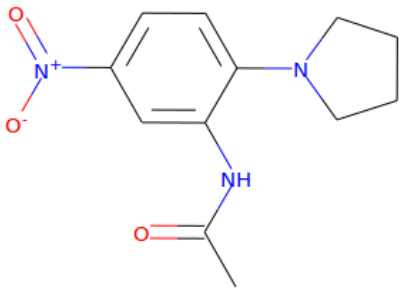	N-(5-nitro-2-pyrrolidin-1-yl-phenyl)acetamide	CC(=O)Nc1cc([N+](=O)[O-])ccc1N1CCCC1
>10 µM<br>2	ZINC14983858	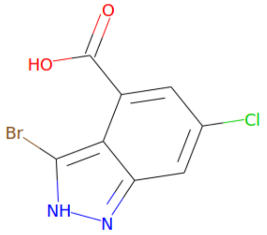	3-Bromo-6-chloro-1H-indazole-4-carboxylic acid	O=C(O)c1cc(Cl)cc2n[nH]c(Br)c12
25 ± 3 nM<br>3	ZINC4143821	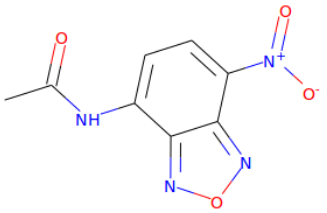	N-(7-nitro-2,1,3-benzoxadiazol-4-yl)acetamide [**NBD-A**]	CC(=O)Nc1ccc([N+](=O)[O-])c2nonc12

>10 µM<br>4	ZINC4176986	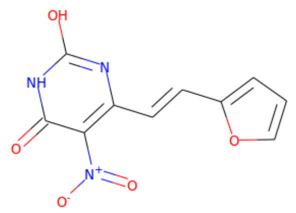	6-[2-(2-furyl)vinyl]-5-nitro-pyrimidine-2,4-diol	O=c1[nH]c(O)nc(/C=C/c2ccco2)c1[N+](=O)[O-]
>10 µM<br>5	ZINC11759621	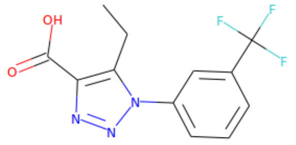	5-ethyl-1-[3-(trifluoromethyl)phenyl]-1H-1,2,3-triazole-4-carboxylic acid	CCc1c(C(=O)O)nnn1-c1cccc(C(F)(F)F)c1
>10 µM<br>6	ZINC12374904	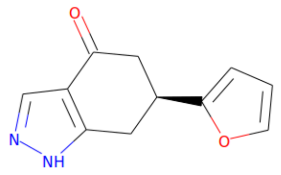	(6S)-6-(2-furyl)-1,5,6,7-tetrahydroindazol-4-one	O=C1C[C@@H](c2ccco2)Cc2[nH]ncc21
>10 µM<br>7	ZINC17142425	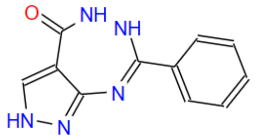	7-phenyl-5,6-dihydropyrazolo[3,4-e]triazepin-4-(2H)-one	O=C1NNC(c2ccccc2)=Nc2n[nH]cc21

>10 μM<br>8	ZINC40168833	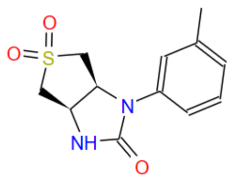	(3aS,6aR)-3-(m-tolyl)-5,5-dioxo-3a,4,6,6a-tetrahydro-1H-thienol[3,4-d]imidazole-2-one	Cc1cccc(N2C(=O)N[C@H]3CS(=O)(=O)C[C@H]32)c1
>10 μM<br>9	ZINC67225235	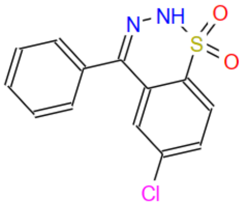	6-chloro-4-phenyl-2H-benzo[e]thiadiazine	O=S1(=O)NN=C(c2ccccc2)c2cc(Cl)ccc21
>10 μM<br>10	ZINC75110699	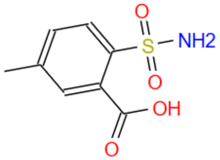	5-methyl-2-sulfamoylbenzoic acid	Cc1ccc(S(N)(=O)=O)c(C(=O)O)c1
>10 μM<br>11	ZINC169744895	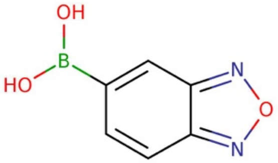	(2,1,3-benzoxadiazol-5-yl)boronic acid	OB(O)c1ccc2nonc2c1

>10 μM<br><br>12	ZINC158674	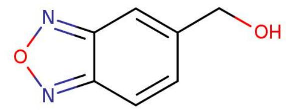	(2,1,3-benzoxadiazol-5-yl)methanol	OCc1ccc2nonc2c1
1.3 ± 0.2 μM<br><br>13	ZINC2166976	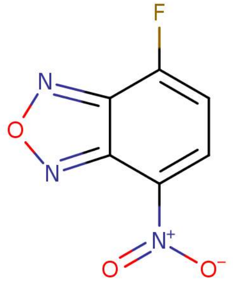	4-fluoro-7-nitro-2,1,3-benzoxadiazole [**NBD-F**]	O=[N+]([O-])c1ccc(F)c2nonc12
62 ± 8 nM<br><br>14	ZINC1311094	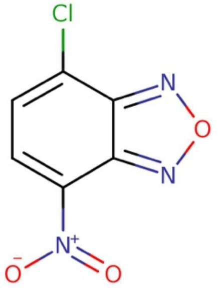	4-chloro-7-nitro-2,1,3-benzoxadiazole (**NBD-Cl**)	O=[N+]([O-])c1ccc(Cl)c2nonc12

5 ± 0.6 µM<br>15	ZINC32951456	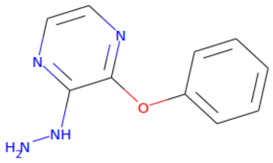	2-hydrazinyl-3-phenoxypyrazine [**HAP**]	NNc1nccnc1Oc1ccccc1
>10 µM<br>16	ZINC6665844	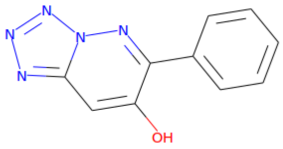	6-phenyl-[1,2,3,4]tetrazolo[1,5-b]pyridazin-7-ol	Oc1cc2nnnn2nc1-c1ccccc1
>10 µM<br>17	ZINC17058363	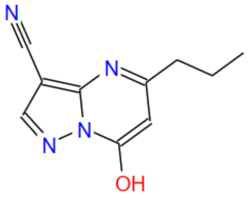	7-oxo-5-propyl-4H,7H-pyrazolo[1,5-a]pyrimidine-3-carbonitrile	CCCc1cc(O)n2ncc(C#N)c2n1
>10 µM<br>18	ZINC14983172	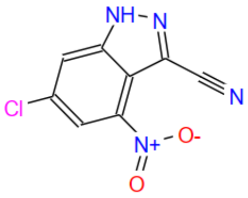	6-Chloro-4-nitro-1H-indazole-3-carbonitrile	N#Cc1n[nH]c2cc(Cl)cc([N+](=O)[O-])c12

>10 μM<br><br>19	ZINC14983946	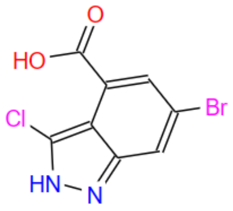	6-Bromo-3-chloro-1H-indazole-4-carboxylic acid	O=C(O)c1cc(Br)cc2n[nH]c(Cl)c1 2

Table S3. Primers used in this study

Name	Usage	Primer sequence (5' to 3')	Enzyme
TbCav-IF	RNAi	CGGGATCCGGTGCACCGTCACAAACTTC	BamHI
TbCav-IR	RNAi	CCCAAGCTTACCCAGTAACAGCACTCGTG	HindIII
TbCav-TF	Tagging	GGCGAATACCAGCCACCACTAGGAACAGATCCC<br>ACCAGTTGGCTTGGTAGCAATGTAAACCGTGGGT<br>CAACCGTAGGTGGACCAACGACAGAATCTCGCA<br>CGAGTAGCGTTATGCCTGCTCCACAGGGACCTAC<br>AGCACCAGAGGGTACCGGGCCCCCCCTCGAG	
TbCav-TR	Tagging	TATACATCGAAACTGGGCTTGTGAACGGATGGTT<br>ATACACATGTGGGCATATATATATAATGGGGGTG<br>CTTCACCCGAGCAAGATTGCATGTCTTCACCTCA<br>CATTTTTTTTCACGCATAAGCTTTGGATAATATC<br>GGTGTGGCGGCCGCTCTAGAACTAGTGGAT	
TbTRPP-TF	Tagging	CGTGAGGACGTGAGAGTGGAAGTGTTGGAGGAC<br>ATCACTGACAGTATAGAAGTACTGCTTCAGCTTG<br>AGGCCACCGAAGTGAGTGCGAGGGAAAAGAAAA<br>CCAGAAGTGATGTTGCGGCAAGTGACAGCAGCG<br>CTGTGAGCGTGGGTACCGGGCCCCCCCTCGAG	
TbTRPP-TR	Tagging	CCACACACCCCCAATTGGTGTGTGAAGAGCAGA<br>CATGCACATTTCCATCTGTGCAGCATGTATTCTC<br>AGGGAGGAAAAAAAAGAAAATAAAAAGTACGC<br>CTTCACCAATGGTAAGTTTCCTGCAAATAACTTA<br>ACCTCCAATGGCGGCCGCTCTAGAACTAGTGGAT	
TbCav-YF	Expression	GACTCACTATAGGGAATATTAAGCTTATGGCTGA<br>GCCGCCACCACCGCAACCACTACG	HindIII
TbCavHA-YR	Expression	CACTGGCGGCCGTTACTAGTGGATCC**CTA***TGCGT*<br>*AATCGGGCACATCGTACGGGTATGCGTAGTCTGGC*<br>*ACGTCGTATGGGTACGCGTAATCAGGCACATCGTAA*<br>*GGGTA*CTCTGGTGCTGTAGGTCCCTGTGGAGC	BamHI
TbCav-HF	Expression	TAGGGAGACCCAAGCTGGCTAGCCCACCATGGC<br>TGAGCCGCCACCACCGCAACCACTACG	NheI
TbCavHA-HR	Expression	TTAAACGGGCCCTCTAGACTCGAG**CTA***TGCGTAA*<br>*TCGGGCACATCGTACGGGTATGCGTAGTCTGGCAC*<br>*GTCGTATGGGTACGCGTAATCAGGCACATCGTAAGG*<br>*GTA*CTCTGGTGCTGTAGGTCCCTGTGGAGC	XhoI

The last letter "F"s or "R"s of the primer names stand for "forward" and "reverse" primers, respectively. The underlined nucleotides indicate the introduced restriction endonuclease sites as described in the "enzyme" column. The italicized or bold nucleotides indicate the introduced 3× HA coding sequence and stop codon in the reverse primer, respectively.

References

(1) Huerta-Cepas, J.; Serra, F.; Bork, P. ETE 3: Reconstruction, analysis, and visualization of phylogenomic data. *Mol Biol Evol* **2016**, *33* (6), 1635-1638. DOI: 10.1093/molbev/msw046.

(2) Guindon, S.; Dufayard, J. F.; Lefort, V.; Anisimova, M.; Hordijk, W.; Gascuel, O. New algorithms and methods to estimate maximum-likelihood phylogenies: assessing the performance of PhyML 3.0. *Syst Biol* **2010**, *59* (3), 307-321. DOI: 10.1093/sysbio/syq010.

(3) Hui, K.; Kwok, T. C.; Kostelecki, W.; Leen, J.; Roy, P. J.; Feng, Z. P. Differential sensitivities of CaV1.2 IIS5-S6 mutants to 1,4-dihydropyridine analogs. *Eur J Pharmacol* **2009**, *602* (2-3), 255-261. DOI: 10.1016/j.ejphar.2008.11.051.

(4) Charnet, P.; Ouadid, H.; Richard, S.; Nargeot, J. Electrophysiological analysis of the action of nifedipine and nicardipine on myocardial fibers. *Fundam Clin Pharmacol* **1987**, *1* (6), 413-431. DOI: 10.1111/j.1472-8206.1987.tb00575.x.

(5) Hirota K, Tsubouchi A, Nakajima-Shimada J, Nara T, Aoki T. Inhibition of *Trypanosoma cruzi* growth in mammalian cells by nimodipine, with low cytotoxicity to host cells. Trop. Med. Health 2004:2, 181-188

(6) Lin, M.; Aladejebi, O.; Hockerman, G. H. Distinct properties of amlodipine and nicardipine block of the voltage-dependent Ca^{2+} channels Cav1.2 and Cav2.1 and the mutant channels Cav1.2/dihydropyridine insensitive and Cav2.1/dihydropyridine sensitive. *Eur J Pharmacol* **2011**, *670* (1), 105-113. DOI: 10.1016/j.ejphar.2011.08.005.

(7) Maya, J. D.; Morello, A.; Repetto, Y.; Tellez, R.; Rodriguez, A.; Zelada, U.; Puebla, P.; Caballero, E.; Medarde, M.; Nunez-Vergara, L. J.; et al. Effects of 3-chloro-phenyl-1,4-dihydropyridine derivatives on *Trypanosoma cruzi* epimastigotes. *Comp Biochem Physiol C Toxicol Pharmacol* **2000**, *125* (1), 103-109. DOI: 10.1016/s0742-8413(99)00096-1.

(8) Johnson, J. D.; Fugman, D. A. Calcium and calmodulin antagonists binding to calmodulin and relaxation of coronary segments. *J Pharmacol Exp Ther* **1983**, *226* (2), 330-334.

(9) Martinuc, J.; Drevensek, G.; Budihna, M. V. Action of mibefradil and lacidipine on the isolated human anterior tibial artery. *Pflugers Arch* **2000**, *440* (5 Suppl), R147-148.

(10) Reimao, J. Q.; Scotti, M. T.; Tempone, A. G. Anti-leishmanial and anti-trypanosomal activities of 1,4-dihydropyridines: In vitro evaluation and structure-activity relationship study. *Bioorg Med Chem* **2010**, *18* (22), 8044-8053. DOI: 10.1016/j.bmc.2010.09.015.

(11) Takenaka, T.; Forster, H.; Epstein, M. Protein kinase C and calcium channel activation as determinants of renal vasoconstriction by angiotensin II and endothelin. *Circ Res* **1993**, *73* (4), 743-750. DOI: 10.1161/01.res.73.4.743.

(12) Rovnyak, G. C.; Atwal, K. S.; Hedberg, A.; Kimball, S. D.; Moreland, S.; Gougoutas, J. Z.; O'Reilly, B. C.; Schwartz, J.; Malley, M. F. Dihydropyrimidine calcium channel blockers. 4. Basic 3-substituted-4-aryl-1,4-dihydropyrimidine-5-carboxylic acid esters. Potent antihypertensive agents. *J Med Chem* **1992**, *35* (17), 3254-3263. DOI: 10.1021/jm00095a023.

(13) Atwal, K. S.; Rovnyak, G. C.; Kimball, S. D.; Floyd, D. M.; Moreland, S.; Swanson, B. N.; Gougoutas, J. Z.; Schwartz, J.; Smillie, K. M.; Malley, M. F. Dihydropyrimidine calcium channel blockers. 2. 3-substituted-4-aryl-1,4-dihydro-6-methyl-5-pyrimidinecarboxylic acid esters as potent mimics of dihydropyridines. *J Med Chem* **1990**, *33* (9), 2629-2635. DOI: 10.1021/jm00171a044.

(14) Nunez-Vergara, L. J.; Squella, J. A.; Bollo-Dragnic, S.; Marin-Catalan, R.; Pino, L.; Diaz-Araya, G.; Letelier, M. E. Isradipine and lacidipine: effects in vivo and in vitro on *Trypanosoma cruzi* epimastigotes. *Gen Pharmacol* **1998**, *30* (1), 85-87. DOI: 10.1016/s0306-3623(97)00077-3.
